# SKI activates the Hippo pathway via LIMD1 to inhibit cardiac fibroblast activation

**DOI:** 10.1007/s00395-021-00865-9

**Published:** 2021-04-13

**Authors:** Natalie M. Landry, Sunil G. Rattan, Krista L. Filomeno, Thomas W. Meier, Simon C. Meier, Sarah J. Foran, Claire F. Meier, Navid Koleini, Robert R. Fandrich, Elissavet Kardami, Todd A. Duhamel, Ian M. C. Dixon

**Affiliations:** 1 Institute of Cardiovascular Sciences, St. Boniface Hospital Albrechtsen Research Centre, 351 Taché Avenue, Winnipeg, MB Canada; 2 Department of Physiology and Pathophysiology, University of Manitoba, Winnipeg, Canada; 3 Department of Human Anatomy and Cell Science, University of Manitoba, Winnipeg, Canada; 4 Rady Faculty of Health Sciences, Max Rady College of Medicine, University of Manitoba, Winnipeg, Canada; 5 Faculty of Kinesiology and Recreation Management, University of Manitoba, Winnipeg, Canada

**Keywords:** Fibroblast, Cardiac fibrosis, Extracellular matrix, Hippo signaling, SKI, TAZ

## Abstract

We have previously shown that overexpression of SKI, an endogenous TGF-β_1_ repressor, deactivates the pro-fibrotic myofibroblast phenotype in the heart. We now show that SKI also functions independently of SMAD/TGF-β signaling, by activating the Hippo tumor-suppressor pathway and inhibiting the Transcriptional co-Activator with PDZ-binding motif (TAZ or WWTR1). The mechanism(s) by which SKI targets TAZ to inhibit cardiac fibroblast activation and fibrogenesis remain undefined. A rat model of post-myocardial infarction was used to examine the expression of TAZ during acute fibrogenesis and chronic heart failure. Results were then corroborated with primary rat cardiac fibroblast cell culture performed both on plastic and on inert elastic substrates, along with the use of siRNA and adenoviral expression vectors for active forms of SKI, YAP, and TAZ. Gene expression was examined by qPCR and luciferase assays, while protein expression was examined by immunoblotting and fluorescence microscopy. Cell phenotype was further assessed by functional assays. Finally, to elucidate SKI’s effects on Hippo signaling, the SKI and TAZ interactomes were captured in human cardiac fibroblasts using BioID2 and mass spectrometry. Potential interactors were investigated in vitro to reveal novel mechanisms of action for SKI. In vitro assays on elastic substrates revealed the ability of TAZ to overcome environmental stimuli and induce the activation of hypersynthetic cardiac myofibroblasts. Further cell-based assays demonstrated that SKI causes specific proteasomal degradation of TAZ, but not YAP, and shifts actin cytoskeleton dynamics to inhibit myofibroblast activation. These findings were supported by identifying the bi-phasic expression of TAZ in vivo during post-MI remodeling and fibrosis. BioID2-based interactomics in human cardiac fibroblasts suggest that SKI interacts with actin-modifying proteins and with LIM Domain-containing protein 1 (LIMD1), a negative regulator of Hippo signaling. Furthermore, we found that LATS2 interacts with TAZ, whereas LATS1 does not, and that LATS2 knockdown prevented TAZ downregulation with SKI overexpression. Our findings indicate that SKI’s capacity to regulate cardiac fibroblast activation is mediated, in part, by Hippo signaling. We postulate that the interaction between SKI and TAZ in cardiac fibroblasts is arbitrated by LIMD1, an important intermediary in focal adhesion-associated signaling pathways. This study contributes to the understanding of the unique physiology of cardiac fibroblasts, and of the relationship between SKI expression and cell phenotype.

**Supplementary Information:**

The online version contains supplementary material available at 10.1007/s00395-021-00865-9.

## Background

Following soft-tissue injury, rapid expansion and remodeling of the extracellular matrix (ECM) is essential for local wound healing response. However, chronic activation of fibroblasts into the hyper-secretory myofibroblast phenotype leads to excess synthesis and deposition of matrix and matrix-associated proteins, including fibrillar collagens types I and III [18, 27], periostin [4, 46], and the cell-associated fibronectin extracellular domain A (ED-A FN) splice variant [29, 45]. Another hallmark of the myofibroblast phenotype is the incorporation of alpha-smooth muscle actin (αSMA) into cytoskeletal actin stress fibers [50], a trait which imparts a contractile quality to the cell. In the heart, post-myocardial infarction (post-MI) wound healing progresses into chronic expansion of the infarct scar, as the heart possesses limited innate regenerative properties. Ensuing myocardial fibrosis and subsequent loss of functional myocardium then contributes to the patient’s decline into heart failure [30]. Despite this reality, there are no effective therapeutic interventions to prevent or heal cardiac fibrosis.

Originally identified as the cellular homolog of the avian Sloan-Kettering virus, SKI is a multi-functional transcriptional co-regulator, commonly regarded as a negative regulator of SMAD-dependent TGF-β signaling [7, 59]. In the nucleus, the SKI associates with Nuclear Co-Repressors (NCoR) and HDAC proteins to form an inhibitory complex [52, 54], believed to confer anti-fibrotic properties. SKI dysfunction is causal to Shprintzen–Goldberg syndrome, a connective tissue disorder similar to Marfan syndrome, which manifests from a loss-of-function mutation in SKI’s SMAD-binding domain [42]. In addition, there is some evidence that SKI also functions in an SMAD-independent manner, specifically via the inhibition of CREB-binding protein (CBP) and the AP-1 complex [10, 61]. Our group has previously reported that ectopic expression of SKI in primary cardiac myofibroblasts reduced the expression of fibrillar collagens and αSMA, which was observed in concert with a decrease in cellular contractility [11, 12]. Furthermore, SKI is dysregulated and sequestered to the cytoplasm in a post-MI model of cardiac remodeling, suggesting that it contributes to fibroblast phenotype regulation [12]. We found that SKI also modulates MMP-9 expression and releases from primary cardiac fibroblasts, indicating that it may also influence the deposition and removal of ECM during post-MI wound healing [31]. As SKI’s effects in cardiac fibroblasts are substantial and robust, it is possible that its functions within the cell extend beyond SMAD inhibition.

As a potential contributor to the regulation of the cardiac fibroblast phenotype, the Hippo signaling pathway was identified as another potential mechanism by which SKI moderates the activation of myofibroblasts. The Hippo pathway is a tumor-suppressor signaling cascade that is composed of core kinases including Macrophage Stimulating 1 or 2 (Mst1/2) and Large Tumor Suppressor 1 or 2 (LATS1/2) [19, 63]. These kinases, along with several scaffolding proteins, regulate the primary nuclear effectors of the pathway, Yes-Associated Protein (YAP) [9] and its paralog, Transcriptional co-Activator with PDZ-binding motif (TAZ, also called WWTR1) [25]. Both YAP and TAZ do not possess any direct DNA-binding domains; however, they strongly associate with TEAD and TEF transcription factors [24, 62] to promote the expression of pro-fibrotic targets such as Connective Tissue Growth Factor (CTGF) and Cysteine-Rich angiogenic factor 61 (CYR61) [8]. When activated, the core Hippo signaling complex inhibits YAP and TAZ by phosphorylation via LATS1/2, followed by cytoplasmic shuttling and proteasomal degradation. In the heart, it has been determined that Hippo signaling plays an important role in cardiac development and homeostasis [22, 57], and considerable effort to investigate its promotion of cardiomyocyte re-entry into the cell cycle for post-MI regeneration. While YAP/TAZ signaling is linked to the pathogenesis of various fibro-proliferative diseases [3, 39, 49], there is limited information regarding the role of Hippo and YAP/TAZ signaling in cardiac fibroblast phenotype regulation in the injured heart.

Herein, we present evidence that SKI activates the Hippo-signaling pathway, and specifically does so to inhibit the pro-fibrotic activity of TAZ, independent of YAP. Our study suggests that SKI dysregulation during post-MI remodeling results in the inhibition of the Hippo kinase cascade, thus allowing fibrosis to persist in the myocardium.

## Materials and methods

### Ethics

All studies presented herein were conducted in accordance with the guidelines and principles of the Canadian Council on Animal Care (CCAC), as well as the Canadian Tri-Council Policy Statement for Ethical Conduct on Research Involving Humans (TCPS 2, 2018). Ethics approval for both animal and human tissue collection was provided by the University of Manitoba’s Office of Research Ethics and Compliance, and Protocol Management and Review Committee. All cardiac surgery patients at St. Boniface General Hospital signed a consent form allowing tissue materials removed and discarded as a normal part of surgery to be used for research purposes, according to the University of Manitoba and St. Boniface General Hospital institutional polices. Based on this, the Research Ethics Board of the University of Manitoba waived the need for individual informed consent by donors and granted permission for use of human tissue from cardiac surgery patients (#H2016:274).

### In Vivo model of myocardial infarction

Young male Sprague–Dawley ranging from 125 to 150 g in mass underwent left anterior descending (LAD) coronary artery ligation or sham surgery as previously described [14]. Animals were randomly sorted into the following timepoints for echocardiography and subsequent tissue harvest: 48 h, 4 days, 1 week, 2 weeks, 4 weeks, and 8 weeks. A total of *n* = 30 sham-operated and *n* = 49 LAD-ligated animals were used to acquire the samples for this study.

Tissues were collected from animals anaesthetized with 3% isoflurane for a minimum of 10 min. Hearts were excised and immediately washed in 1X PBS, after which they were dissected into discrete sections for future analyses. The sham-operated hearts were separated into right (RV) and left (LV) ventricles, while the LAD ligated hearts were separated into RV, viable LV, and infarcted LV (scar). Tissues intended for histology were frozen fresh in optimal cutting temperature (OCT) compound (VWR International, Radnor, PA; #95,057–838) and flash-freezing in a dry ice-ethanol bath. Freshly isolated tissue and frozen blocks were immediately stored at – 80 °C until used.

### Protein isolation from frozen tissue

Frozen tissue samples were crushed using a pre-chilled mortar and pestle, while submerged in an excess volume of liquid nitrogen. The crushed tissue (and liquid nitrogen) was then decanted into a sterile 15 mL conical tube containing 1 mL tissue lysis buffer (125 mM Tris, pH 7.4; 1% SDS; 5% glycerol; 1X protease inhibitor cocktail (Sigma-Aldrich, #P8340), 10 mM NaF, 1.0 mM Na_3_VO_4_, and 1.0 mM EGTA) per 100 mg of tissue. The samples were then incubated on ice for 1 h, with periodic vortexing to ensure complete lysis. Following 10 s of sonication, the lysate was transferred to QIAshredder columns (QIAgen, Hilden, Germany) and centrifuged according to the manufacturer’s recommended conditions. The flow-through was collected and stored at – 80 °C until use.

### Preparation of tissue culture surfaces

Silicone elastic tissue culture plates and coverslips bearing an elastic modulus of 5 kPa were purchased from Excellness Biotech (Lausanne, Switzerland). Prior to use, surfaces were sterilized 100% isopropanol and thoroughly dried. Surfaces were then coated overnight at 37 °C using an excess volume of 10 μg/mL porcine gelatin type A diluted in PBS, and used at a concentration of 2 µg/cm^2^. The following day, the gelatin solution was aspirated, and cells were plated immediately in sufficient culture medium.

### Primary rat cardiac fibroblast isolation and culture

Rat primary cardiac fibroblasts were isolated, as previously described [32]. Male Sprague–Dawley rats weighing 101–125 g were anaesthetized with a ketamine–xylazine cocktail (100 mg/kg ketamine; 10 mg/kg xylazine) administered intraperitoneally. After verifying the loss of limb reflexes, heparin (6 mg/kg) was administered intravenously via the saphenous artery. Hearts were immediately excised and placed in 1:1 Dulbecco’s Modified Eagle’s medium/Ham’s F12 nutrient mixture (DMEM/F12, Gibco # 11,320-033). The hearts were cannulated via the aorta on a Langendorff apparatus and then subject to retrograde perfusion with DMEM/F12 for 5 min, followed by Minimum Essential Medium, Spinner’s Modification (S-MEM, Gibco # 11,380-037) for another 5 min to flush out calcium and promote cell dissociation. To digest the tissue, the hearts were then perfused with S-MEM supplemented with 600 U/mL collagenase type II (Worthington Biochemical Corporation, Lakewood, NJ; #CLS-2) with recirculation for 25 min at 37 °C.

For quiescent fibroblast culture, the digested tissue was incubated at 37 °C, 5% CO_2_ for 10 min, and then neutralized with 10 mL of Ham’s F-10 medium (F-10, Gibco, # 11,550-043) supplemented with 2% fetal bovine serum (FBS) and 100 U/mL penicillin–streptomycin. The tissue was further dissociated by trituration and the final cell suspension was gravimetrically passed through a 40 μm sterile cell strainer (Thermo Fisher Scientific, Waltham, MA). The cells were pelleted by centrifugation at 200×*g* for 5 min, and re-suspended in complete cell culture medium. Fibroblasts were allowed to adhere to prepared elastic surfaces for 3 h at 37 °C, 5% CO_2_. Adherent cells were then briefly washed twice with 1X PBS supplemented with penicillin–streptomycin, and fresh complete culture medium was added. For the following 3 days, the cultures were once again washed, and the growth medium was replaced. Cells were used for experimentation 4-day post-plating.

For activated myofibroblast culture, digested tissue was incubated as described above, but was neutralized with DMEM/F12 supplemented with 10% FBS and penicillin–streptomycin. Cells were allowed the adhere for 2 h at 37 °C, 5% CO_2_ on conventional plastic surfaces prior to brief washing in 1X PBS and replacing the complete culture medium. Cells were allowed to proliferate until ~ 75–80% confluence prior to passaging. Experiments using activated rat myofibroblasts were passaged once (P1).

### Adenoviral constructs and in vitro infection

All adenoviral constructs were designed to overexpress the human gene product under control of the CMV promoter. The viruses overexpressing LacZ, HA-tagged SKI, and the MYC-BioID2 fusion proteins, were generated by our lab using Adeno-X Expression System (Takara Bio Inc., Kusatsu, Japan), as per the manufacturer’s instructions. The FLAG-tagged constitutively active YAP[5SA] and MYC-tagged TAZ[4SA] viruses were designed by our lab and generated by VectorBuilder Inc. (Chicago, IL, USA). Viral DNA vectors were sequenced for the presence of the transgene insert at The Centre for Applied Genomics (Hospital for Sick Children, Toronto, ON) prior to amplification.

To overexpress a given protein of interest, cells were serum-starved overnight (~ 16 h) in F-10 medium supplemented with penicillin–streptomycin. The following day, the cells were infected with a vector-dependent multiplicity of infection (MOI) of 20–50 in serum-free F-10 medium. Viral infection was allowed to proceed for approximately 36 h prior to harvesting for analysis.

### Primary cell treatment with small molecule inhibitors

P1 primary rat cardiac fibroblasts were seeded onto stiff plastic culture plates at about 20% confluency (~ 7.0 × 10^5^ cells/10 cm dish). Once the cells reached 40–50% confluency, they were serum-starved overnight in DMEM/F12. The culture medium was then replenished (still serum-free), and supplemented with one of the following small-molecule inhibitors: MG132 (1 µM; Sigma-Aldrich; #M7449), GS143 (1 µM; Tocris Bioscience, Bristol, UK; #5636), D4476 (500 nM; Selleckchem, Houston, TX; #S7642). Control plates were treated with DMSO alone. Cells were pre-treated with the compounds for 3 h, after which they were infected with either Ad-LacZ or Ad-HA-SKI (as described above; MOI of 50) and cells were harvested 24 h post-infection.

### siRNA-mediated gene knockdown

First-passage (P1) cardiac myofibroblasts were seeded at 1.0 × 10^4^ cells in each well of a 6-well dish. Cells were left to adhere overnight in DMEM/F12 supplemented with 10% FBS and 100 U/mL of penicillin–streptomycin. The cells were then starved overnight in serum-free, antibiotic-free DMEM (Gibco, # 10,564–011). The following day, cells were transfected for 24 h with 50 nM of either a non-targeting siRNA pool (Dharmacon, Lafayette, CO) or a 4-oligo pool targeting the gene of interest using Lipofectamine RNAiMax (ThermoFisher) as per the manufacturer’s protocol. If the assay was performed in conjunction with protein overexpression, the following day the medium was changed, and the cells were infected with the appropriate viral construct. Whole cell lysates were collected for analysis after approximately 36 h of viral overexpression.

### Protein isolation from cell cultures

Fibroblasts cultured on elastic surfaces were trypsinized and pelleted prior to lysis. Cell pellets were lysed with RIPA lysis buffer (1% NP-40; 0.5% NaDOC; 0.1% SDS; 50 mM Tris–HCl, pH 7.4; 150 mM NaCl; 10% glycerol; 1 mM EDTA) supplemented with protease inhibitors (Sigma, P8430) and phosphatase inhibitors (10 mM NaF; 1 mM Na_3_VO_4_; 1 mM EGTA). Lysates were incubated on ice for 30 min with periodic vortexing; this was followed by brief sonication and centrifugation at 16 000 × *g* for 15 min at 4 °C. Cleared lysates were then transferred to fresh microcentrifuge tubes for total protein determination by BCA assay (Thermo Scientific, # 23,225). Cytoplasmic and nuclear cell fractionation was achieved using the Pierce NE-PER™ Extraction reagents (Thermo Scientific, #78,833). All samples were kept at –20 °C for short-term use, or –80 °C for long-term storage (Table 1).

**Table 1:** siRNA oligo pools

Target	Species	Accession	Pool (Dharmacon™ Cat#)
*Lats1*	*R. norvegicus*	NM_001134543.2	M-080189–01-0005
*Lats2*	*R. norvegicus*	NM_001107267.1	M-087043–01-0005
*Limd1*	*R. norvegicus*	NM_001112737.2	L-081750–02-0005
*Ski*	*R. norvegicus*	XM_017593893	L-099478–02-0005
*Wwtr1 (Taz)*	*R. norvegicus*	NM_001024869.1	M-088521–01-0005
*Non-targeting pool*	*H. sapiens, M. musculus, R. norvegicus*	n/a	D-001810–10-05

### Immunoblotting

SDS-PAGE of 20–30 μg of protein was performed on 4–15% gradient reducing gels. Proteins were then transferred 4 °C onto PVDF membranes at 4 °C and total protein loading was measured prior to blotting using Ponceau S (Alfa Aesar, Haverhill, MA; #J63139) staining, or after blotting using Pelikan 17 black india ink (Thomas Scientific, Swedesboro, NJ; #C861L76). Blots were incubated with primary antibodies (listed in Table 2) overnight at 4 °C with gentle shaking. Corresponding HRP-conjugated secondary antibodies (Jackson ImmunoResearch, West Grove, PA) were applied at a 1:5000–1:20 000 dilution for 1 h at room temperature. Antibody detection was done using ECL substrate, and protein bands were visualized on blue X-ray film (Mandel Scientific, Guelph, ON). Protein expression was measured by relative densitometry using Quantity One analysis software (version 4.6.9; Bio-Rad).

**Table 2:** List of primary antibodies used for immunoblotting

Antibody target	Host	Manufacturer	Catalogue number
ED-A (Cellular) Fibronectin	Mouse	MilliporeSigma, Burlington, MA, USA	MAB1940
αSMA	Mouse	MilliporeSigma, Burlington, MA, USA	A2547
Periostin	Rabbit	Abcam, Cambridge UK	ab14041
Vimentin	Mouse	Abcam, Cambridge UK	ab8069
YAP	Rabbit	Cell Signaling, Danvers, MA, USA	14,074
TAZ/WWTR1	Rabbit	Cell Signaling, Danvers, MA, USA	83,669
GAPDH	Mouse	Cell Signaling, Danvers, MA, USA	97,166
LIMD1	Rabbit	Abcepta Inc, San Diengo, CA, USA	AP13132b
LATS1	Rabbit	Cell Signaling, Danvers, MA, USA	3477
LATS2	Rabbit	Cell Signaling, Danvers, MA, USA	5888
HA-tag	Rabbit	Rockland Immunochemicals, Limerick PA, USA	600–401-384
MYC-tag	Rabbit	Cell Signaling, Danvers, MA, USA	2278
NCoR1	Rabbit	Cell Signaling, Danvers, MA, USA	5948
SKI	Rabbit	Novus Biologicals, Littleton, CO, USA	NBP2-94,105
TEAD3	Rabbit	Cell Signaling, Danvers, MA, USA	13,224

### RNA isolation and quantitative PCR

Cardiac fibroblasts were harvested by trypsinization and centrifugation at 200 × *g* for 5 min. Column-based total RNA isolation was performed using the PureLink RNA Mini kit (Invitrogen, Carlsbad, CA; # 12,183,025) following the manufacturer’s instructions. DNase treatment and cDNA synthesis were performed on 50 ng of total RNA (Maxima First Strand cDNA Synthesis Kit for RT-qPCR, with dsDNase; Thermo Scientific; #K1672), and the final product was diluted to 200 µL sterile TE buffer (10 mM Tris–HCl, 1 mM EDTA, pH 8.0) before use.

Gene expression was assayed using Luna® Universal qPCR Master Mix (New England Biolabs, Ipswich, MA; #M3003). All reactions were performed in technical triplicates on a QuantStudio 3 Real-Time PCR System (Applied Biosystems, Foster City, CA). All samples from resting (P0, 5 kPa plates) fibroblasts were normalized to endogenous *Ywhaz* expression, while samples from activated myofibroblasts (P1, plastic plates) were normalized to *Hprt1*. qPCR primer pairs and their targets are listed in Table 3.

**Table 3:** qPCR primers

Gene	Species	Gene product	Accession	Forward primer (5′- 3′)	Reverse primer (5′-3′)
*Acta2*	*R. norvegicus*	alpha-Smooth Muscle Actin	NM_031004.2	AGATCGTCCGTGACATCAAGG	TCATTCCCGATGGTGATCAC
*Ccn2 (Ctgf)*	*R. norvegicus*	Connective Tissue Growth Factor	NM_022266.2	CAAGCTGCCCGGGAAAT	CGGTCCTTGGGCTCATCA
*Col1a1*	*R. norvegicus*	Collagen Type I Alpha 1 Chain	NM_053304.1	TGCTCCTCTTAGGGGCCA	CGTCTCACCATTAGGGACCCT
*Col1a2*	*R. norvegicus*	Collagen Type I Alpha 2 Chain	NM_053356.1	TGACCAGCCTCGCTCACAG	CAATCCAGTAGTAATCGCTCTTCCA
*Col3a1*	*R. norvegicus*	Collagen Type III Alpha 1 Chain	NM_032085.1	GGTTTCTTCTCACCCTGCTTC	GGTTCTGGCTTCCAGACATC
*Cthrc1*	*R. norvegicus*	Collagen Triple Helix Repeat Containing 1	NM_172333.2	CTATCTGGACCAAGGAAGCCC	CAGATGGCCACGTCTACCAG
*Fn1*	*R. norvegicus*	Fibronectin (ED-A Splice Variant)	NM_019143.2	ACTGCAGTGACCAACATTGACC	CACCCTGTACCTGGAAACTTGC
*Limd1*	*R. norvegicus*	LIM Domain containing protein 1	NM_001112737.2	AACAGGCCTTTGGTCCACTG	GCCTCATATCCCAGACTCGAA
*Hprt1*	*R. norvegicus*	Hypoxanthine–Guanine Phosphoribosyltransferase	NM_012583.2	CTCATGGACTGATTATGGACAGGAC	GCAGGTCAGCAAAGAACTTATAGCC
*Postn*	*R. norvegicus*	Periostin	NM_001108550.1	GCTTCAGAAGCCACTTTGTC	CGCCAACTACATCGACAAGG
*RelA*	*R. norvegicus*	NFkB subunit RelA (Trancription Factor p65)	NM_199267.2	TTCCCTGAAGTGGAGCTAGGA	CATGTCGAGGAAGACACTGGA
*Tcf21*	*R. norvegicus*	Transcription Factor 21	NM_001032397.1	CATTCACCCAGTCAACCTGA	CCACTTCCTTTAGGTCACTCTC
*Wwtr1*	*R. norvegicus*	WW domain containing Transcription Regulator 1 (TAZ)	NM_001024869.1	ACCTGGCTGTAGTGTGATGC	CCAGGCAATGATTAAGCGGC
*Yap1*	*R. norvegicus*	Yes-Associated Protein 1	NM_001034002.2	CAGACAACAACATGGCAGGAC	CTTGCTCCCATCCATCAGGAAG
*Ywhaz*	*R. norvegicus*	Tyrosine 3-monooxygenase/Tryptophan 5-monooxygenase Activation protein Zeta	NM_013011.3	TTGAGCAGAAGACGGAAGGT	GAAGCATTGGGGATCAAGAA

### Plasmid expression vectors

Mutation of human YAP and TAZ to promote nuclear translocation was performed using the QuickChange II Site-Directed Mutagenesis Kit (Agilent Technologies, Santa Clara, CA; #200,523), with some modifications to the manufacturer’s protocol, as described by Zheng et al*.* [64]. Both wild-type genes were cloned into pcDNA3 prior to mutagenesis. All plasmid constructs were sequenced for confirmation of the mutations (YAP S127A, TAZ S89A) and complete coverage of the insert.

### Luciferase reporter assay

Proximal promoters for the human *Col1a1* (1.4 kb) and *Col3a1* (1.5 kb) genes were generated from human genomic and subcloned into the pGL4.1[*Luc2*] vector. NIH-3T3 fibroblasts were seeded in 6-well dishes 24 h prior to transfection at a density that would yield ~ 60% confluency. Wells were co-transfected using jetPRIME DNA transfection reagent (Polyplus Transfection, Illkirch, France; #114–15) with 500 ng pGL4.1[*Luc2*] promoter–reporter plasmid and 500 ng expression vector for 48 h. Cells transfected with empty expression vector with the corresponding promoter construct served as background control samples. Promoter activity was assayed using a Luciferase Reporter Assay Kit I (PromoCell GmbH, Heidelberg, Germany; # PK-CA707-30,003–1) and luciferase activity was quantified on a GloMax Multi + Multimode Plate Reader (Promega, Madison, WI). All samples were assays in triplicates and relative promoter activity was normalized to total protein concentration for the corresponding sample.

### Collagen gel contraction assay

Three-dimensional collagen gels were prepared by mixing 16 mL of chilled collagen solution (PureCol® Type I bovine collagen; Advanced BioMatrix, Sand Diego, CA), with 2 mL of sterile 10X PBS. While keeping the solution on ice, the pH was adjusted to 7.4 using sterile 0.1 M NaOH, and the volume was brought up to 20 mL with sterile water. Gels were cast in a 24-well plate by adding 600 µL solution to each well and allowing them to solidify at 37 °C, 5% CO_2_ overnight. The following day, P0 fibroblasts cultured on 5 kPa elastic surfaces were passaged and seeded on the collagen matrices at a density of 1.5 × 10^4^ cells per well and allowed to adhere for 24 h. The gels were then released from the wells using a circular cutting tool and immediately infected with their corresponding adenoviral vector, or treated with 4 ng/mL recombinant human TGF-β_1_ (Cell Signaling, #8915) as a positive control. Images were taken immediately following treatment, and subsequently every 24 h for a total of 72 h post-treatment. Gel contraction was estimated by measuring the surface area of the top of the gel using ImageJ image processing software [44].

### Wound healing assay

Silicone inserts (Ibidi, Martinsried, Germany; #81,176) were placed into the wells of a porcine gelatin-coated 24-well 5 kPa elastic tissue culture dish (Excellness, as described above). 70 uL of freshly isolated primary rat cardiac fibroblast cell suspension (~ 5.0 × 10^4^ cells/mL) was added to each chamber of the insert, and 0.5 mL of F-10 medium supplemented with 2% FBS was added to the area surrounding the insert. The culture medium was changed every day for the following 3 days, after which the cells were allowed to reach 80% confluency within the insert chambers. The inserts were then removed, and the cells were infected with an MOI of 50 of their corresponding adenoviral constructs in serum-free F-10 medium. Light microscopy images were captured at 6, 12, and 18 h post-infection, after which percent surface area coverage was calculated using ImageJ software.

### Isolation of human atrial cardiac fibroblasts

Human primary cardiac fibroblasts were obtained from male patients undergoing elective, isolated coronary artery bypass graft (CABG) surgery. Cells were isolated as previously described [41]. In brief, freshly isolated tissue was finely minced and placed in culture dishes containing basal medium supplemented with 10% fetal bovine serum (FBS), 100 units/mL penicillin and 100 μg/mL streptomycin. Cells were allowed to migrate from the explants for approximately 10 days prior to passaging, at which point they were maintained in complete cardiac fibroblast growth medium (FGM™, Lonza Group AG, Basel, Switzerland). Cells were passaged at least two times (P2) prior to using in experiments, and P3 cells were frozen for future use and used for BioID2 experiments.

### BioID2 assay and mass spectrometry analysis

BioID2 fusion proteins were generated from the myc-BioID2-MCS plasmid and Adeno-X Adenoviral Expression System 1 (Takara Bio Inc., Kusatsu, Japan). The myc-BioID2-MCS vector was a gift from Kyle Roux (Addgene plasmid # 74,223; http://n2t.net/addgene:74223; RRID:Addgene_74223). In vitro biotinylation assays were performed on primary human cardiac fibroblasts; cells passaged three times (P3) were used in BioID2 assays, and were considered as activated myofibroblasts.

Once at approximately 40% confluency, cells were switched to serum-free F10 medium, and infected with an MOI of 20 of either: Ad-myc-BioID2-Empty, Ad-myc-BioID2-hSKI, or Ad-myc-BioID2-hTAZ. After 24 h, the medium was supplemented with 20 μM biotin from a freshly prepared 100X stock diluted in warm culture medium. Finally, after further incubation for 18 h, the medium was removed and the cells were briefly washed twice with room-temperature 1X phosphate buffered saline (PBS). Cells were lysed with 500 μL lysis buffer (1% NP-40; 0.5% deoxycholate; 0.2% SDS; 2% Triton X-100; 50 mM Tris HCl, pH 7.4; 150 mM NaCl; 1 mM EDTA; 10% glycerol; supplemented with 1X Sigma protease inhibitor cocktail P8340). Samples were briefly sonicated, and then, samples were then diluted with an equal volume of 50 mM Tris HCl, pH 7.4 and incubated overnight on a rotator at 4 °C with 400 μL of magnetic streptavidin beads (#S1420S, New England Biolabs, Ipswich, MA) that had been pre-washed for 10 min in lysis buffer. The following day, the beads were washed once in 2% SDS for 10 min, followed by four times for 10 min each in 50 mM Tris HCl, pH 7.4. The beads were resuspended in 1 mL wash buffer, and 20% was saved for analysis by Western blot. The remaining beads were resuspended in 250 μL of 50 mM NH_4_HCO_3_ and sent for mass spectrometry analysis at the Manitoba Centre for Proteomics and Systems Biology (Winnipeg, MB, Canada).

Samples isolated by BioID2 pulldown were subject to tryptic digestion, and digested peptide solutions for each biological replicate was subject to two technical replicate analyses on a Q Exactive™ HF-X Orbitrap mass spectrometer (Thermo Scientific, Waltham, MA) in standard tandem MS/MS with data-dependent acquisition. The protein identification parameters were as follows: minimum fragment M/Z of 100; precursor mass tolerance of ± 10 ppm; fragment mass error of 0.02 Da, and a maximum *E* value of 0.01. Fixed modifications for oxidation of methionine and tryptophan, deamination of asparagine and glutamine, and carboxyamidomethylation of cysteine were also included in the analytical parameters. Peptides were compared against the SwissProt human protein database and were identified and quantified using X!tandem (Alanine, 2017.02.01). Proteins with fewer than 3 spectral counts, common background and contaminating proteins (e.g., keratins, histones, trypsin) were excluded from the resulting data. Technical replicates were then combined for interactome analysis.

Significance of interaction between bait (SKI or TAZ) and prey was determined using probabilistic scoring via the Significance of Analysis of Interactome express (SAINTexpress) algorithm [53]. Using the Contaminant Repository for Affinity Purification (CRAPome.org) [37], SAINTexpress was applied to the data sets using user-uploaded (untreated and empty BioID2 vector) controls, incorporation of iRefIndex data, and using all replicates per bait. SAINT scores and fold-change values for both SKI and TAZ are displayed in Supplemental Tables 1 and 3 respectively. Proteins included in the final interactomes had a fold change of ≥ 3 compared to empty-BioID2 and untreated controls, and obtained a SAINT score ≥ 0.5. Our rationale for lowering the SAINT score was due to the fact that there exists no repository for primary human cardiac fibroblast protein interactions, and the vast majority of proximity-labelling data that is publicly available has been gathered in cell lines. The resulting data were then formatted for use in Cytoscape (version 3.7.1) to generate interaction network graphics, and Gene Ontology (GO) and pathway analyses were performed using the STRING database (version 11.0) and WikiPathways (wikipathways.org) [28]. Finally, novel interactions were confirmed by repeated affinity capture and probing for targets by immunoblotting. Data were generated using cells isolated from *n* = 4 biological samples.

The mass spectrometry proteomics data have been deposited to the ProteomeXchange Consortium via the PRIDE partner repository with the data set identifier PXD018246. To access the data, the following login information may be used: Username: reviewer00088@ebi.ac.uk Password: A7zMZkwM. A copy of the data has also been included in the online data supplement.

### F/G actin isolation and fractionation

Cardiac myofibroblasts were cultured and treated in 10 cm dishes, and harvested at ~ 50–60% confluency. Cells were washed twice in pre-warmed 1X PBS to remove any excess medium and serum. After removing as much PBS as possible, F and G actin were isolated using a G-actin/F-actin in vivo Assay Kit (Cytoskeleton Inc., Denver, CO; #BK037), as per the manufacturer’s directions. Immunoblotting was performed with pan-actin antibody (1:1000; Cell Signaling; #4968), and peroxidase-conjugated goat anti-rabbit secondary antibodies (1:10 000; Jackson ImmunoResearch).

### Fluorescence immunohistochemistry (IHC-F)

Frozen OCT-preserved tissues were cut into 6 µm sections using a cryostat microtome and mounted on glass slides which were kept in a Coplin jar on ice. Sections were allowed to air dry and adhere to the slides for 30 min while remaining on ice. The slides were then washed twice for 5 min with warm PBS to remove as much OCT as possible.

Tissue sections were fixed in 4% paraformaldehyde in 1X PBS (pH 7.4) for 10 min, after which they were washed in neutralizing buffer (0.4 M Glycine in 1X PBS, pH 7.4) twice for 5 min each. Sections were then permeabilized for 15 min at room temperature in 0.1% Triton X-100 in PBS. Non-specific binding sites were then blocked for 30 min in 5% Normal Goat Serum (Invitrogen, # 50-197Z) in PBS. Primary antibodies were diluted in 1% Bovine Serum Albumin (Alfa Aesar, #J64655) in PBS with 0.05% Triton X-100 to their appropriate concentration. The following antibodies were used—YAP (1:100; Cell Signaling; #14,074), TAZ/WWTR1 (1:100; Cell Signaling; #4883S), and Periostin (1:100; LSBio, Seattle, WA; #LS-B10443). Sections were incubated in YAP or TAZ primary antibody solutions overnight (~ 18 h) at 4 °C. After multiple 5-min washes in PBS, Alexa Fluor-conjugated secondary antibodies (488 goat anti-rabbit, Invitrogen, #A27034) were applied at a 1:500 dilution in the same dilution buffer as the primary antibodies, and incubated at room temperature for 90 min. After several 5-min washes, the sections were incubated with Periostin primary antibody conjugated to the Alexa Fluor 594 fluorophore (Lightning-Link®; Abcam; #ab269822) at room temperature for 90 min. After several washes over a 45-min period, glass coverslips were applied with 20 µL mounting medium with DAPI (Invitrogen, #P36971) per section. Slides were allowed to cure overnight at room temperature, and sections were imaged with a Zeiss LSM 5 Pa microscope; images were acquired and processed using AxioVision Microscopy software (Zeiss, rel. 4.8).

### Fluorescence immunocytochemistry (ICC-F)

Cells were seeded at a low (~ 10–15%) confluency onto either glass coverslips in 6-well dishes, or elastic (5 kPa) silicone coverslips (ExCellness) in 35 mm dishes, coated with porcine gelatin type A. After appropriate treatments, the cells were briefly washed in PBS and fixed in warm 4% paraformaldehyde in PBS, pH 7.4 for 15 min. After another two brief washes in PBS, cells were permeabilized and non-specific binding sites were blocked for 1 h in 5% normal goat serum (Invitrogen) in PBS with 0.1% Triton X-100. Primary antibodies diluted in 1% bovine serum albumin (BSA) in PBS were applied overnight at 4 °C. The following primary antibodies were used: αSMA (1:200; Sigma; #A2547), YAP (1:100; Cell Signaling; #14,074), TAZ/WWTR1 (1:100; Cell Signaling; #4883S), ED-A fibronectin (1:100; Millipore Sigma; #MAB1940), LIMD1 (1:100; R&D Systems, Minneapolis, MN; #MAB8494), SKI (1:100; Santa Cruz; #sc-33693). The following day, the cells were washed three times in PBS and incubated with Alexa Fluor fluorophore-conjugated secondary antibodies at 1:100 dilution for 1 h at room temperature: 488 rabbit anti-mouse (Invitrogen, #A27023), 647 rabbit anti-mouse (Invitrogen, #A27029), 488 goat anti-rabbit (Invitrogen, #A27034). For the samples stained for αSMA, after the initial incubation with secondary antibodies, F-actin was stained using a 1:500 dilution of rhodamine-phalloidin (R415; Invitrogen) in PBS for 30 min. The coverslips were mounted on glass slides using mounting medium with DAPI (Invitrogen, #P36971) and allowed to cure at room temperature for 24 h. Coverslips were then sealed, and cells were imaged using a Zeiss LSM 5 Pa microscope as described above.

### Data analysis and statistics

Statistical analyses and graphic data representations were primarily generated using GraphPad Prism 8 (version 8.1.2; May 2019). All data are represented by the mean ± standard deviation, unless otherwise indicated in the figure legends. Individual biological replicates (n values) are defined as originating from experiments involving cells or tissue from one single animal or human donor. Biological replicates from assays involving cell lines were defined as having originated from cells from different passages. Data distribution was assessed by the Shapiro–Wilk test. Non-parametric grouped data analyses were performed using Kruskal–Wallis tests. Grouped data with normal distribution (*P* > 0.05 Shapiro–Wilk test) were analyzed by one- or two-way ANOVA with Tukey’s post hoc test for multiple comparisons or Dunnet’s post hoc test when performing multiple comparisons to the controls only. In vivo data were analyzed with right (RV) and left ventricles (LV) as separate groups. Sham RV and MI RV were compared using two-way ANOVA with Sidak correction, while sham LV, viable MI viable LV and MI Scar LV were analyzed by two-way ANOVA with Dunnett’s correction. Experiments involving only one control and one test condition were analyzed by *t* tests. Significance was recorded if *P* < 0.05.

## Results

### TAZ activates the synthetic cardiac myofibroblast phenotype

**Fig. 1:**
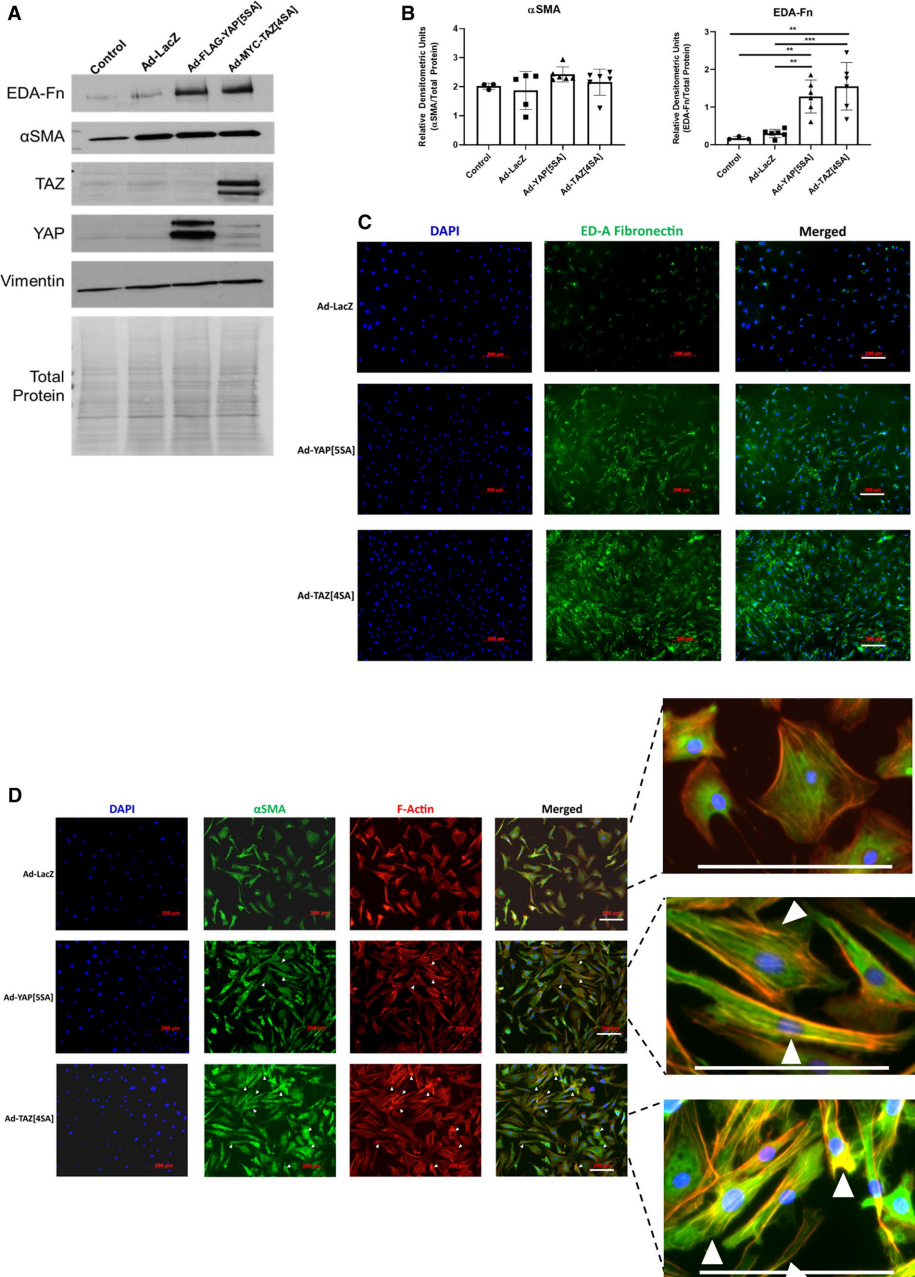
YAP and TAZ induce cardiac myofibroblast marker expression. **a** Immunoblotting of whole cell lysates from unpassaged (P0) primary rat cardiac fibroblasts cultured on 5 kPa elastic silicone tissue culture surfaces coated with gelatin. Cells were treated with adenoviral constructs overexpressing constitutively active forms of YAP (Ad-FLAG-YAP[5SA]), TAZ (Ad-MYC-TAZ[4SA]), or Ad-LacZ controls for approximately 36 h. **b** Quantification of densitometric measurements represented in panel A (*n* = 3 untreated controls; *n* = 6 biological replicates per test condition). Data are presented at mean ± SD. *P < 0.05 versus untreated and Ad-LacZ infected controls. **c** P0 primary rat cardiac fibroblasts cultured on 5 kPa elastic silicone coverslips were infected with constitutively active Ad-YAP[5SA], Ad-TAZ[4SA], or Ad-LacZ for 36 h prior to fixation and for indirect immunofluorescence detection of fibronectin extracellular domain splice variant A (ED-A FN; green); nuclei were counterstained with DAPI (blue). Scale bar = 200 µm. **d** P0 primary rat cardiac fibroblasts treated as described for panel C, with indirect immunofluorescence detection of alpha-Smooth Muscle Actin (αSMA; green) and F-actin (phalloidin staining; red). White arrows indicate cells with greater inclusion of αSMA into F-actin stress fibers. Data shown for C and D are representative of *n* = 3 biological replicates

**Fig. 2:**
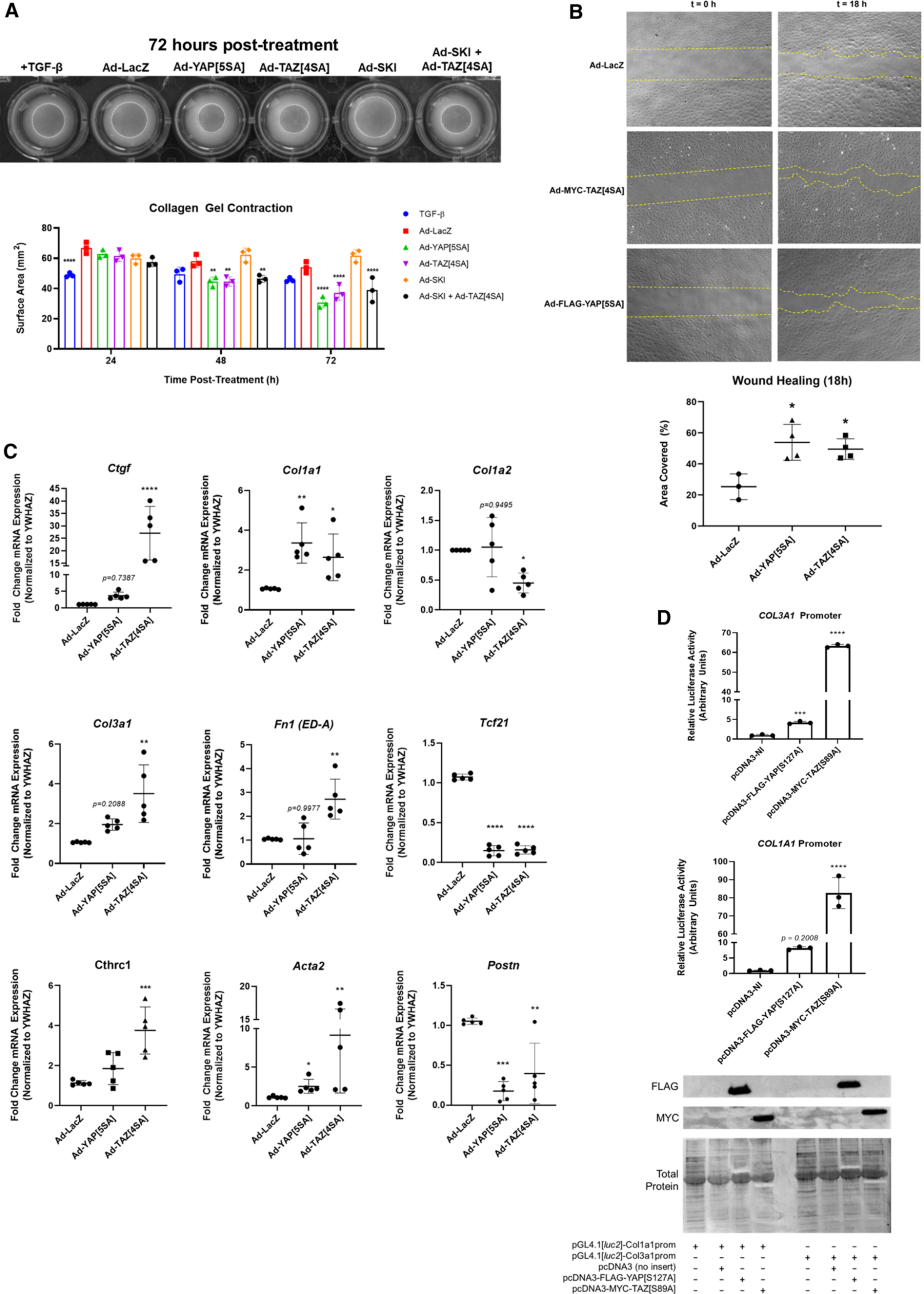
Cardiac myofibroblast gene expression and physiology are enhanced by TAZ expression**. a** P0 rat cardiac fibroblasts seeded on two-dimensional collagen matrices were assayed for gel contraction following infection with Ad-YAP[5SA], Ad-TAZ[4SA], Ad-SKI, a combination thereof, or with Ad-LacZ control. Treatment with recombinant human TGF-β_1_ served as a positive control. Images were captured at 24, 48, and 72 h post-treatment; the image in the upper panel was captured at the 72-h timepoint. The lower panel displays the quantification of the surface area of the top of the collagen matrices, measured in mm^2^. Data shown are representative of *n* = 4 biological replicates, where **P < 0.01, ****P < 0.0001 when compared to Ad-LacZ infected controls. **b** Rat cardiac fibroblasts seeded into inserts with a defined cell-free gap on 5 kPa elastic silicone surfaces were infected with either YAP[5SA], TAZ[4SA], or LacZ-expressing adenoviral constructs at an MOI of 50. Wound healing rate was assessed as percent surface area covered by cells at 18 h post-infection. Data are representative of *n* = 4 biological replicates, with *P < 0.05 when compared to Ad-LacZ infected controls. **c** P0 primary rat cardiac fibroblasts cultured on 5 kPa elastic silicone tissue culture surfaces coated with gelatin were transduced with adenoviral constructs overexpressing Hippo effectors, Ad-YAP[5SA] or Ad-TAZ[4SA], or Ad-LacZ control. mRNA was isolated 48 h post-infection, and analyzed by qRT-PCR, with *n* = 5 biological replicates per condition. Data are reported as mean fold-change with respect to Ad-LacZ infected controls. *P < 0.05, **P < 0.01, ***P < 0.001, ****P < 0.0001 when compared to Ad-LacZ infected controls. **d** NIH-3T3 fibroblasts were transfected with either an empty pcDNA3-NI (NI, no insert), -YAP[S127A], or -TAZ[S89A] expressing vectors in conjunction with a luciferase reporter–promoter plasmid (pGL4.1[*luc2*]) containing either the human Collagen 1α1 (*COL1A1*) or 3α1 (*COL3A1*) promoter. Luciferase activity was assayed 48 h post-transfection, and normalized to pcDNA3 transfected controls. Data are representative of *n* = 3 biological replicates (3 technical replicates each), with ***P < 0.001, ****P < 0.00001 when compared to pcDNA3-transfected controls. All data (A-D) are reported as mean ± SD

When plated on inert elastic substrata, primary cardiac fibroblasts retain a quiescent, fibroblast-like phenotype for several days in two-dimensional culture [32]. To determine whether YAP and/or TAZ are contributors to cardiac fibroblast activation, we assessed whether either can overcome culture conditions which promote the fibroblast phenotype. Primary rat cardiac fibroblasts were treated with adenoviruses which expressed constitutively active forms of human YAP and TAZ which resist phosphorylation and are expressed as predominantly nuclear proteins. Serine residues were modified to alanine as previously reported [13, 35]: for YAP, S61A, S109A, S127A, S164A, and S397A (YAP[5SA]) [47]; for TAZ, S66A, S89A, S117A, and S311A (TAZ[4SA]) [33]. This was done for two reasons: first, the baseline expression of both YAP and TAZ in cardiac fibroblasts cultured on soft substrates is very low and nearly undetectable on Western blots; second, overexpressed wild-type YAP and TAZ are subject to rapid phosphorylation and degradation, yielding results consistent with a dominant-negative mutant. In primary cardiac fibroblasts, overexpression of the constitutively active forms of both YAP and TAZ yielded similar increases in ED-A FN protein expression; the change was slightly more pronounced with TAZ, but the results varied among biological replicates (Fig. 1a–c). In addition, when *Taz* was targeted with siRNA, its elimination also led to the inhibition of EDA-FN expression (Supplemental Fig. 1). Conversely, there was no apparent change in overall αSMA protein expression with overexpression of either YAP or TAZ when compared to Ad-LacZ infected controls. While total αSMA expression was unchanged, its inclusion in cytoplasmic stress fibers was increased with both YAP and TAZ overexpression, despite the cells being plated on 5 kPa elastic culture surfaces (Fig. 1d). Furthermore, two-dimensional gel contraction assays indicated that there was indeed an increase in the contractility of both YAP[5SA] and TAZ[4SA]-overexpressing fibroblasts, when compared to LacZ and SKI-overexpressing controls (Fig. 2a). It was also observed that when overexpressed in conjunction with TAZ[4SA], SKI’s ability to inhibit the contractile myofibroblast phenotype was nearly completely abrogated; this suggests that SKI’s mechanism to regulate TAZ is dependent on its phosphorylation state. In addition, the wound-healing rate of unpassaged (P0) primary cardiac fibroblasts was increased twofold with both active YAP and TAZ overexpression, as demonstrated by in vitro wound healing assays (Fig. 2b).

Quantitative PCR further confirmed the activation state of primary cardiac fibroblasts treated with either YAP[5SA] or TAZ[4SA] (Fig. 2c). Initial investigations probed for *Ctgf*, *Acta2* (αSMA), and *Fn1* (ED-A splice variant) and revealed that TAZ overexpression in primary cardiac fibroblasts resulted in an acute increase in both *Ctgf* and *Fn1* transcription. While both YAP and TAZ demonstrated at least a twofold increase in *Acta2* transcription, the increase showed greater variance with TAZ. We next probed *Tcf21*, a marker of quiescent resident cardiac fibroblasts which has been shown to decrease in expression during fibrogenesis [17, 26]. Active YAP and TAZ overexpression markedly reduced *Tcf21* transcription, compared to LacZ-overexpressing controls. We also probed for periostin (*Postn),* but did not observe any changes in its transcription. We have found that *Postn* is not a reliable marker for in vitro fibroblast activation in the absence of exogenous TGF-β1 treatment (data not shown). Finally, we examined collagen transcription, specifically for fibrillar monomers 1α1, 1α2 and 3α1. With both YAP and TAZ activation, *Col1a1* transcription was increased, and yet only TAZ significantly increased *Col3a1* expression. In contrast to this was *Col1a2*, which showed no transcriptional modulation with YAP, but was found to slightly decrease with TAZ overexpression. In concert with these findings, Collagen Triple Helix Repeat-Containing 1 (*Cthrc1*) transcript expression was also increased with YAP and TAZ activation, suggesting that these factors indeed contribute to myofibroblast activation and extracellular matrix remodelling. To further examine the role of YAP/TAZ on fibrillar collagen transcription, luciferase assays for the human *COL1A1* and *COL3A1* promoters was performed in NIH-3T3 fibroblasts (Fig. 2d). Upon induction of active YAP expression, the activity of the *COL1A1* and *COL3A1* promoters was increased by approximately eightfold and fourfold, respectively. A stark contrast was observed with active TAZ induction, which yielded over 10 times greater luciferase activity from both promoters, when compared to active YAP. Overall, these data suggest that while both YAP and TAZ do induce a more myofibroblast-like phenotype, TAZ may play a greater role to the incorporation of αSMA into stress fibers and the increased production of fibrillar collagens.

### TAZ expression increases during post-MI fibrogenesis

**Fig. 3:**
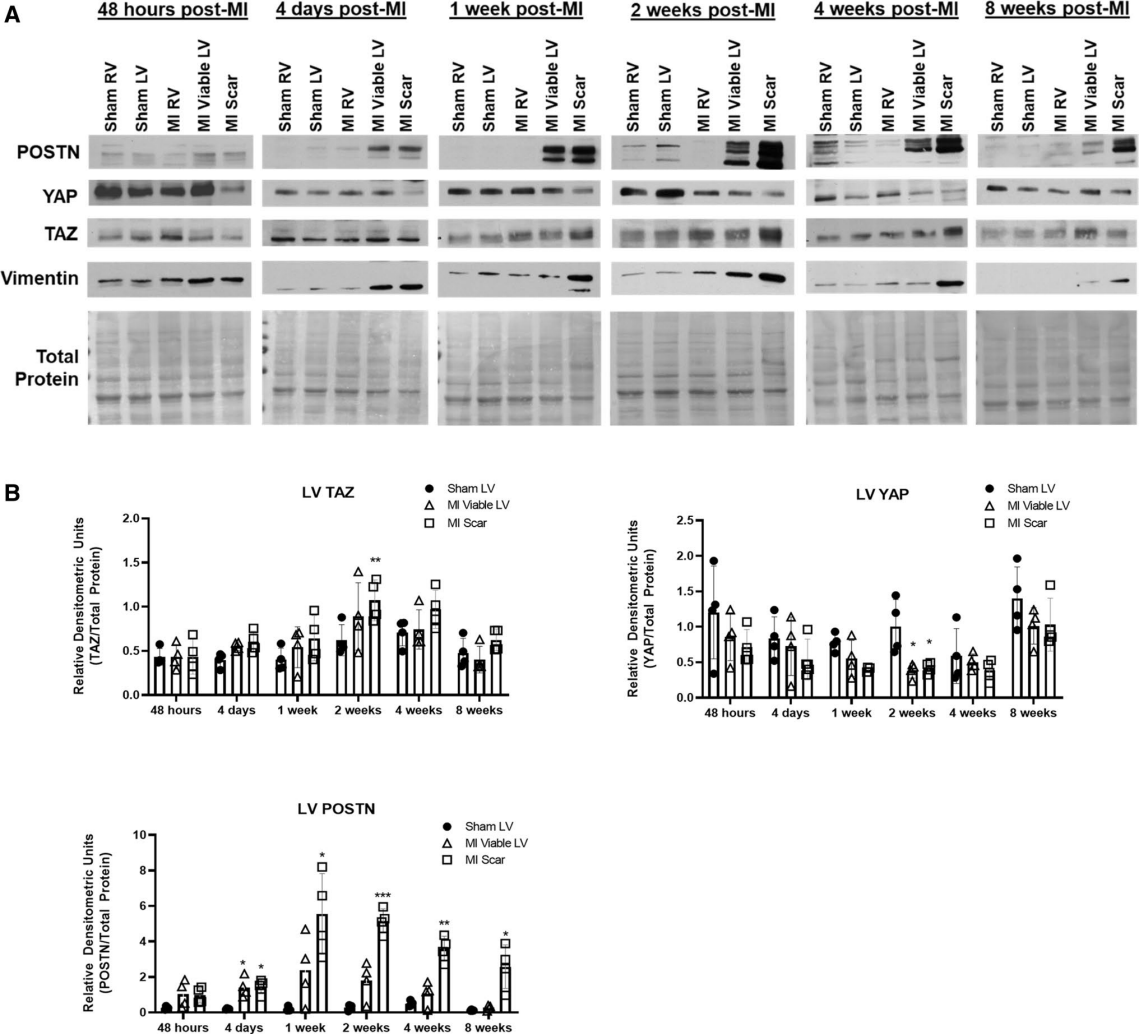
YAP and TAZ expression during post-MI fibrogenesis, in vivo. **a** Immunoblotting of whole tissue lysate from male Sprague–Dawley rats subject to left anterior descending (LAD) coronary artery ligation or sham operation. Hearts were excised at various timepoints, spanning 48 h to 8-week post-ligation, and tissue from left (LV) and right (RV) ventricles were isolated for analysis. **b** Quantification of densitometric measurements of data shown in A. Data are representative of experiments originating from *n* = 4 to 6 animals per timepoint, and are reported as the mean ± SD. *P < 0.05, **P < 0.01, ***P < 0.001, when compared to that tissue’s (RV or LV) corresponding sham animals

**Fig. 4:**
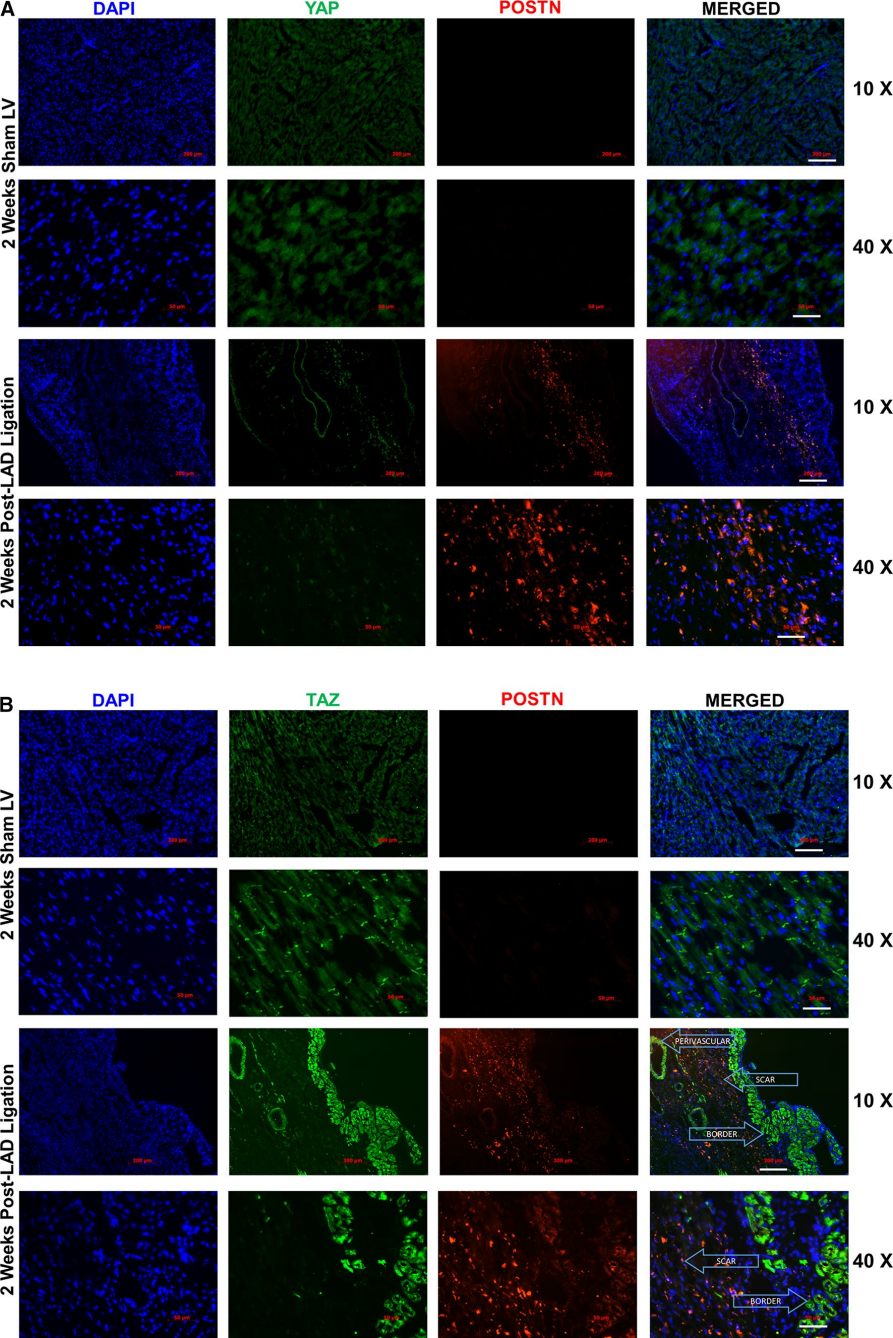
TAZ expression is increased in the surviving noninfarcted myocardium adjacent to the infarct scar and in the infarct scar following MI. Indirect immunofluorescence of LV scar at 2-week post-MI (LAD ligation) or sham operation. Sections were probed for YAP (A panels, green), or TAZ (B panels, green) and extracellular periostin (POSTN—red) for identification of areas containing remodeling tissues. Perivascular medial tissues and infarct scar are indicated with arrows. Periostin staining was apparent in the infarct scar in 2-week post-LAD ligation group. TAZ staining was observed in the Z-line regions of myocytes of noninfarcted control heart sections, and in the perivascular space, surviving myocytes of the border zone myocardium and in the infarct scar. Nuclei were counterstained with DAPI (blue). Scale bars = 200 µm at 10X magnification, and 50 µm at 40X magnification. Images are representative of *n* = 3 biological replicates

To interrogate the role of YAP/TAZ in cardiac fibrogenesis, we implemented a rat post-MI model of acute replacement fibrosis to observe their expression over time. Tissue was collected from rats subject to left anterior descending (LAD) coronary ligation or sham surgery at the following timepoint post-infarct: 48 h, 4 days, 1 week, 2 weeks, 4 weeks, and 8 weeks. In addition, heart tissue was further isolated into individual components for examination: sham left (LV) and right (RV) ventricles, along with LAD ligated RV, viable LV, and LV scar. Using periostin as a marker of fibroblast activation and active matrix remodelling, and vimentin as an indicator of mesenchymal cells, we probed for YAP and TAZ individually at each timepoint. Immunoblotting revealed that TAZ expression peaks at the 2-week timepoint in concert with periostin and vimentin (Fig. 3a), and remains above baseline levels until 4-week post-MI. In sections used for immunofluorescence studies, we found some punctate expression of TAZ and strong periostin staining in the infarct scar. We cannot definitively conclude that the increase in TAZ expression is originating from activated myofibroblasts, as we observed only punctate staining in the expanding infarct scar (Fig. 4b and Supplemental Fig. 5). We also observe TAZ staining in the perivascular medial space and in surviving cardiomyocytes of the myocardium bordering the infarct scar. Furthermore, our data show punctate periostin expression the infarct scar (Fig. 4b and Supplemental Fig. 5). Conversely, YAP expression remains relatively unchanged among all timepoints; however, there is a modest decrease at 2-week post-MI, and an upward trend in the later phases of post-MI remodeling (Fig. 3a, b). When probing tissue sections by immunofluorescence 2-week post-MI, we found that YAP expression shifts from cardiomyocyte-specific expression to reduced levels in infarct scar and border zone tissues (Fig. 4a and Supplemental Fig. 5). between infarcted and sham-operated LV tissue (Fig. 4a, top panels), but is increased in expression in the vascular media. There is sparse YAP staining within the infarcted LV, and periostin is localized to the central, maturing portion of the infarct scar. TAZ expression is markedly enriched in the border zone tissue with punctate staining in the infarct scar (Fig. 4b; Supplemental Fig. 5). There was also significant TAZ staining in the perivascular media and adventitia, although co-staining in areas which are periostin-positive was seldom seen. In sham-operated tissue, TAZ was largely expressed at intercalated discs within cardiomyocytes, and this distinct characteristic feature was subject to redistribution upon infarction (Fig. 4b). It was not evident with YAP or TAZ whether nuclear localization of endogenous protein was altered during post-MI remodeling. Both YAP and TAZ have altered myocardial expression during post-MI remodeling, and we note the presence of some TAZ staining in the infarct scar, with greater staining in the medial perivascular space and surviving cardiomyocytes of the border zone (Fig. 4b and Supplemental Fig. 5). We also observed marked punctate staining of periostin in the infarct scar.

**Fig. 5:**
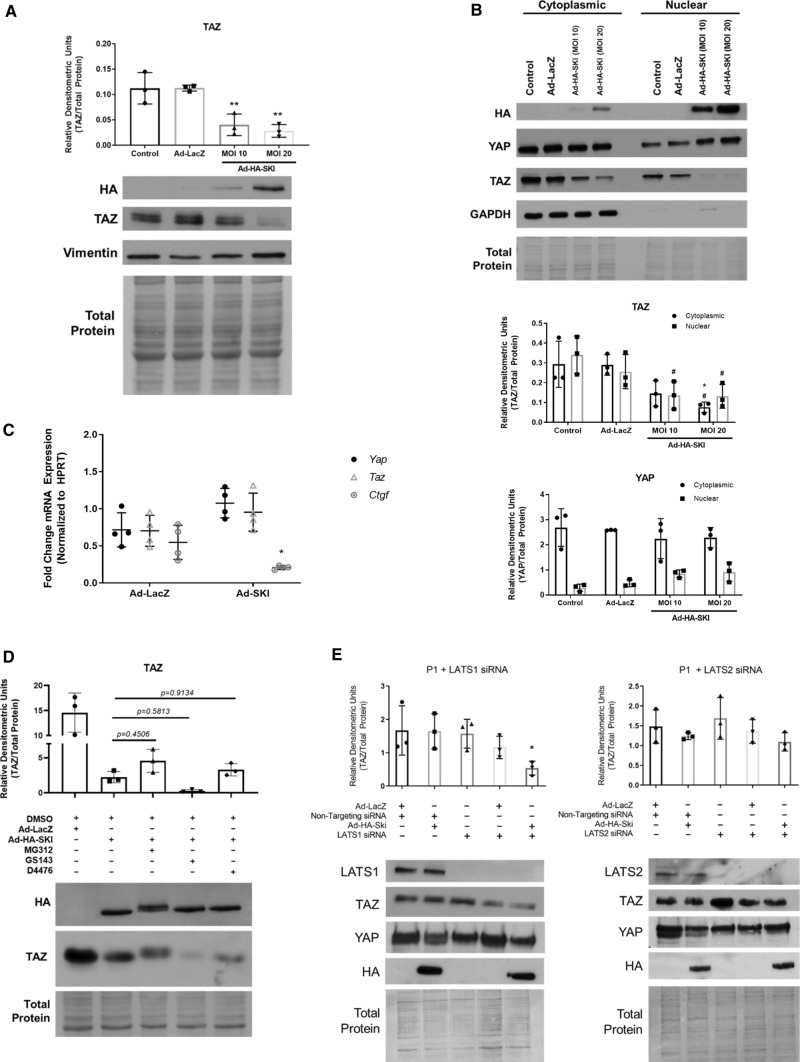
SKI induces proteasomal degradation of TAZ, but not YAP. First-passage (P1) primary rat cardiac myofibroblast were cultured on stiff plastic surfaces and infected with SKI overexpressing adenovirus (Ad-HA-SKI) at a low and high MOI (10 and 20, respectively) for 36 h prior to harvesting. **a** Whole cell lysates were probed by immunoblotting. Data are representative of *n* = 3 biological replicates, where **P < 0.01 when compared to non-treated and Ad-LacZ infected controls. **b** Immunoblotting of nuclear and cytoplasmic subcellular fractions, showing an enrichment of nuclear-localized SKI. Data are representative of *n* = 3 biological replicates, where *P < 0.05 when compared to Ad-LacZ infected controls and ^#^*P* < 0.05 when compared to untreated controls. **c** Gene expression was assayed by qRT-PCR, specifically targeting *Yap* and *Taz*, as well as their genetic target, *Ctgf*. Data are representative of *n* = 4 biological replicates, where *P < 0.05 when compared to Ad-LacZ infected controls for the given genetic target. **d** P1 rat cardiac fibroblasts were pre-treated with either MG132 (1 µM), GS143 (1 µM), or D4476 (500 nM) for 3 h prior to infection with either Ad-HA-SKI or Ad-LacZ control for 24 h. Data are representative of *n* = 3 biological replicates. **e** Prior to infection with SKI-expressing adenovirus, P1 rat cardiac myofibroblasts where transfected with siRNA targeting *Lats1* and *Lats2* kinases for 24 h. Cells were subsequently harvested after 36 h of viral infection. Data are representative of *n* = 3 biological replicates for each condition, where *P < 0.05 compared to cells treated with non-targeting siRNA and Ad-LacZ. All data are displayed (A-E) as the mean ± SD

### SKI induces proteasomal degradation of TAZ, but not YAP

After establishing TAZ as an important element in cardiac fibroblast activation and post-MI remodeling, we determined the effects of SKI on the Hippo pathway by overexpressing it in primary cardiac myofibroblasts. Fibroblasts isolated from healthy rat myocardium were passaged once (P1) to induce the myofibroblast phenotype, and subsequently infected with adenovirus expressing SKI. Immunoblotting of whole cell lysates and subcellular fractions revealed that the overexpressed SKI is predominantly nuclear and that SKI specifically effects a sharp downregulation of TAZ protein expression (Fig. 5a, b). In contrast, a modest upregulation of YAP expression (NS) was observed with SKI overexpression. As SKI is known to be a genetic co-repressor, we sought to rule out that this resulting attenuation in TAZ expression was controlled at the transcriptional level. qPCR analyses of SKI-overexpressing myofibroblasts revealed that both *Yap* and *Taz* transcription were unaffected by ectopic SKI. However, *Ctgf*, a known genetic target of YAP/TAZ, exhibited a threefold reduction in transcription; this further bolstered the hypothesis that SKI regulates TAZ at the level of the protein (Fig. 5c). Finally, when examining *Taz* and *Ctgf* transcription after RNAi-induced knockdown of *Ski* in P0 fibroblasts cultured on soft substrates, the absence of SKI was insufficient in promoting further activation of the myofibroblast phenotype upon treatment with recombinant TGF-β_1_ (Supplemental Fig. 3b). Untreated cells expressed low levels of *Ctgf*, which markedly increased with TGF-β_1_ treatment, but this increase in expression was unaffected by *Ski* knockdown. Moreover, the introduction of TGF-β_1_ did not change *Taz* expression, and this was also unaffected when combined with *Ski-*targeting siRNA. The lack of transcriptional response to *Ski* knockdown suggests that SKI is indeed not implicated in regulating *Taz* transcription. These findings also confirm that SKI is only one part of a complex regulatory mechanism which governs cardiac fibroblast response to TGF-β_1_ stimulation, and that the pleiotropic pathway has multiple avenues—mechanosensory, extracellular, and intracellular—by which it affects response to injury.

To better elucidate the mechanism by which SKI downregulates TAZ protein expression, we treated SKI-overexpressing primary cardiac myofibroblasts with various small molecule inhibitors to probe various points in the Hippo signaling cascade. We first treated cells with the pan-acting MG132 proteasome inhibitor, which yielded modest results in rescuing SKI-mediated TAZ degradation (Fig. 5d). Next, we inhibited the β-TrCP1 E3 ubiquitin ligase, a known regulator of YAP/TAZ expression, using GS143. In contrast to MG132, the downregulation of TAZ protein expression was apparently exacerbated by the inhibition of β-TrCP1. Lastly, we sought to inhibit casein kinase 1 (CK1), which works subsequent to LATS1/2 kinases to form the TAZ phosphodegron in canonical Hippo signaling [34]. Using the CK1 inhibitor, D4476, we observed TAZ degradation with SKI overexpression. The results did not coincide with MG132 treatment, and this is likely due to the pleiotropic nature of CK1, and that it may not be necessary for all instance of phosphorylation-dependent proteasomal degradation of TAZ. This, in concert with the inability of SKI to prevent TAZ[4SA]-mediated cell contraction (Fig. 2a), indicates that SKI indirectly modulates the phosphorylation state and thus the stability of TAZ.

LATS1/2 function in relation to SKI expression was examined in vitro by selectively reducing either LATS1 or LATS2 expression using RNA interference (RNAi). Using siRNA pools, either LATS1 or LATS2 expression was knocked down in primary cardiac myofibroblasts with attending ectopic SKI expression (Fig. 5b, e). SKI overexpression (MOI 10 or 20) in baseline conditions, was associated with downregulated TAZ protein levels (without LATS1 or LATS2 knockdown—Fig. 5b). LATS2 knockdown was associated with “protection” of TAZ expression in the presence of SKI overexpression (Fig. 5e panel 2). Knocking down LATS1 alone did not protect the SKI-mediated downregulation of TAZ. We, therefore, suggest that LATS1 is not required for SKI-mediated degradation of TAZ. We also note in the BioID2 experiment that only LATS2 appeared within in the TAZ interactome, but LATS1 did not—thus TAZ does not interact with LATS1 (Fig. 7c). Furthermore, we noted that the inclusion of nonspecific siRNA also was associated with a loss of TAZ knockdown, and we attribute this to be experimental artifact. From this, we conclude that SKI regulates Hippo signaling in cardiac myofibroblasts by activating LATS2, and this de-repression may then allow for its kinase activity to specifically target TAZ for phosphorylation and degradation (Fig. 9). The apparent destabilizing of TAZ protein contributes to SKI’s inhibition of the myofibroblast phenotype.

### SKI modulates actin cytoskeleton dynamics in cardiac myofibroblasts

**Fig. 6:**
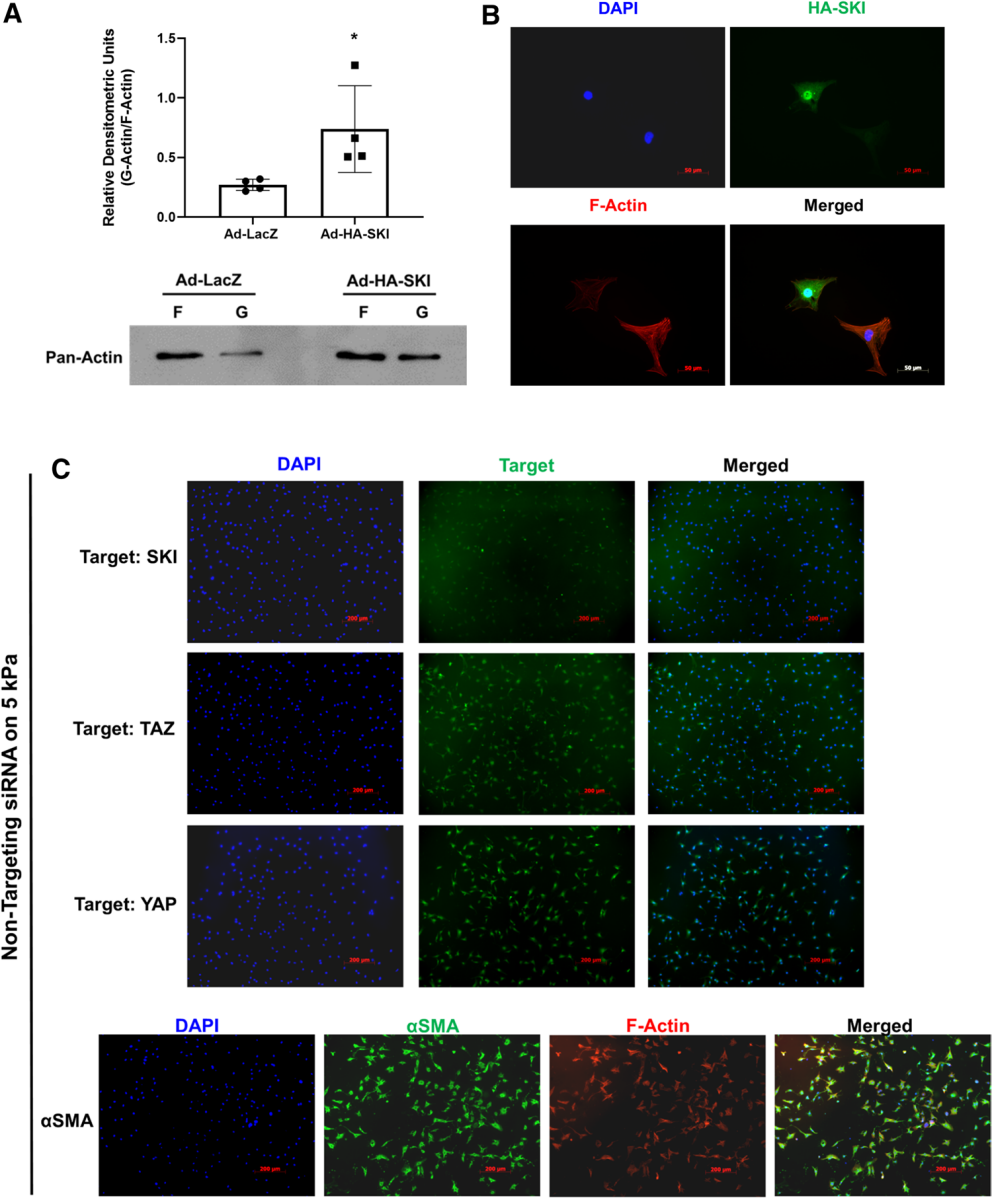

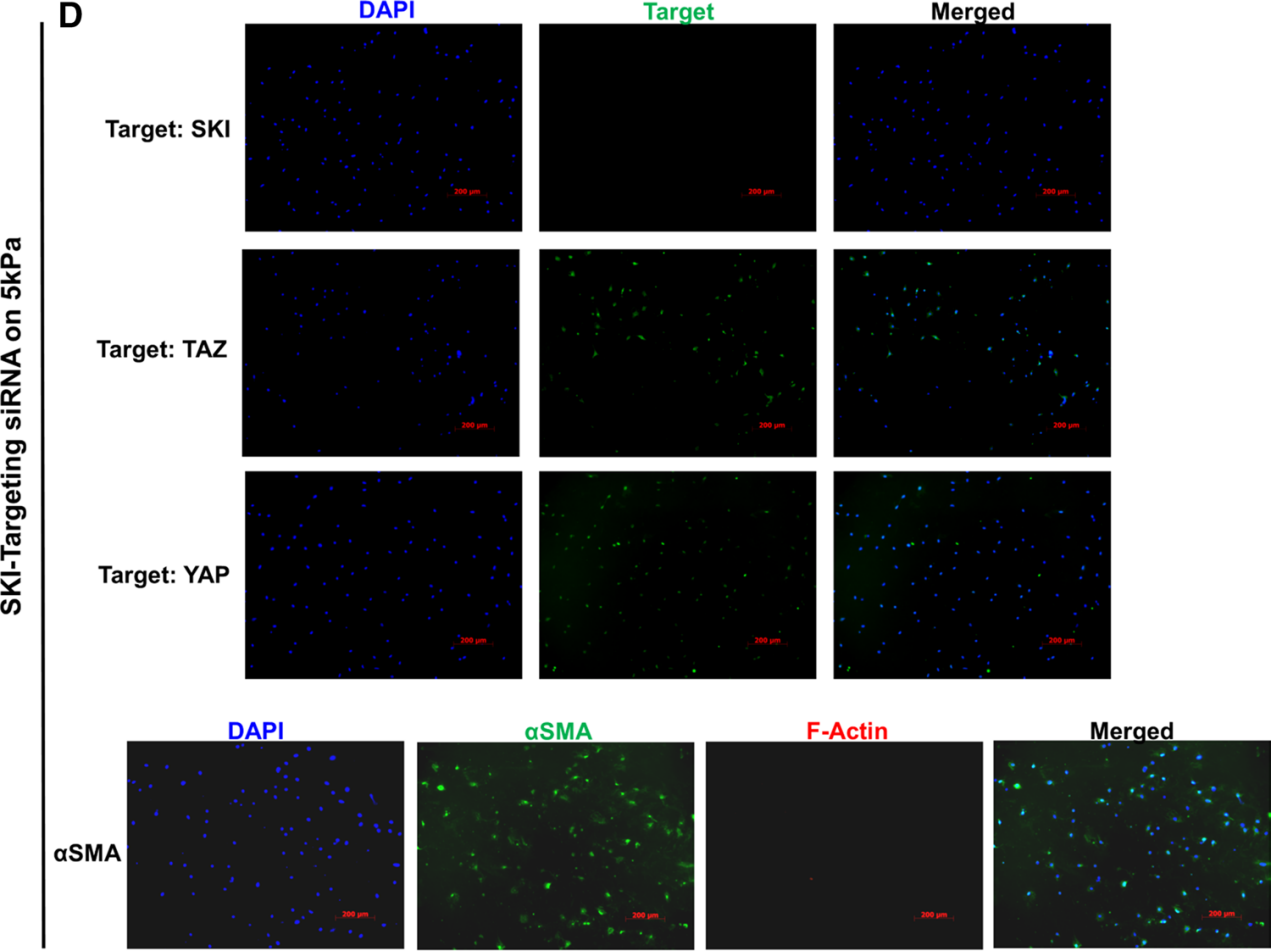
F-Actin polymerization is modulated by SKI expression. First-passage (P1) primary rat cardiac myofibroblasts were infected with SKI-expressing adenovirus (Ad-HA-SKI) or LacZ-expressing control (Ad-LacZ) for 36 h prior to harvesting and isolation of F-actin and soluble G-actin. **a** Equal volumes of each F- or G-actin isolate from one culture dish was separated by SDS-PAGE and immunoblotted with pan-actin antibody. Data are reported as the ratio of G-actin to F-actin in a given sample. *P < 0.05 when compared to Ad-LacZ infected control. **b** HA-SKI overexpressing P1 rat cardiac myofibroblasts were cultured on glass coverslips for 48 h prior to fixation. Cells were probed by indirect immunofluorescence for HA-SKI (green) and F-actin (red), with nuclei counterstained with DAPI (blue). Scale bar = 50 µm. Data shown are representative of *n* = 3 biological replicates, with 2 technical replicates each. **c**, **d** Unpassaged primary cardiac fibroblasts were cultured on 5 kPa compressible silicone surfaces and treated with 50 nM siRNA pools targeting *Ski* (D.) or non-targeting control pools (C.) for 48 h in serum-free F10 culture medium. Cultures were then treated with 10 ng/mL recombinant human TGF-β1 for another 24 h. Cells were then fixed and probed for YAP, TAZ, or αSMA (green) and F-actin (phalloidin; red), with DAPI nuclear counterstaining (blue). Images shown are representative of *n* = 3 biological replicates, with 2 technical replicates for each

We previously showed that SKI downregulates myofibroblast markers in vitro [31]; however, it remains unknown as to whether SKI may influence the effects of the mechanotransduction on stiff substrate. This is salient, as the Hippo pathway is intimately linked to focal adhesions and the actin cytoskeleton, in that it is activated when the cell experiences less mechanical tension and there is a decrease in F-actin stress fibers [15]. Using primary rat cardiac myofibroblasts cultured on stiff substrates, we overexpressed SKI and compared F- and soluble free G-actin ratios to those in Ad-LacZ infected controls. Using cytoskeletal subcellular fractionation and immunoblotting, it was found that SKI significantly reduces relative F-actin levels, and increases free G-actin, even in the presence of high mechanical tension (Fig. 6a). Similar results were observed in immunofluorescence assays, where rhodamine–phalloidin staining was greatly reduced in myofibroblasts overexpressing SKI (Fig. 6b). In addition, siRNA-mediated knockdown of endogenous *Ski* yielded unremarkable results when P0 cardiac fibroblasts were cultured on stiff plastic substrates and treated with exogenous TGF-β1 (Supplemental Fig. 4). However, when the knockdown was carried out on 5 kPa compressible surfaces, there was nearly complete elimination of F-actin, as evidenced by the absence of phalloidin staining, when compared to non-targeting siRNA controls (Fig. 6c, d). Thus the Hippo pathway may be activated by a decrease in F-actin polymerization, and SKI is apparently required to mediate some processes which regulate the synthesis and breakdown of actin fibres. The results indicate that the absence of SKI on a soft matrix yields comparable results to the activation of SKI on a stiff matrix, and the pathophysiological implications likely point to a link between SKI dysregulation and matrix remodelling. These data, along with the evidence corroborating SKI’s effects on Hippo pathway components, indicate that SKI promotes the cardiac fibroblast phenotype by a multifaceted antagonism of TAZ activity.

### The SKI and TAZ interactomes overlap in cardiac fibroblasts

As SKI and TAZ are not known to be binding partners, and SKI’s effects on the Hippo pathway remained elusive, we chose to perform BioID2 assays on both SKI and TAZ to elucidate whether SKI activates LATS2 in a direct or indirect manner. Using BioID2 fusion bait proteins expressed by adenoviral vectors, we captured the SKI and TAZ interactomes in primary human cardiac fibroblasts. We analyzed data from two biological replicates for each bait, and four controls consisting of untreated cell lysate to rule out endogenously biotinylated protein, as well as empty BioID2 to rule out proteins that may have a natural affinity for the BirA ligase.

**Fig. 7:**
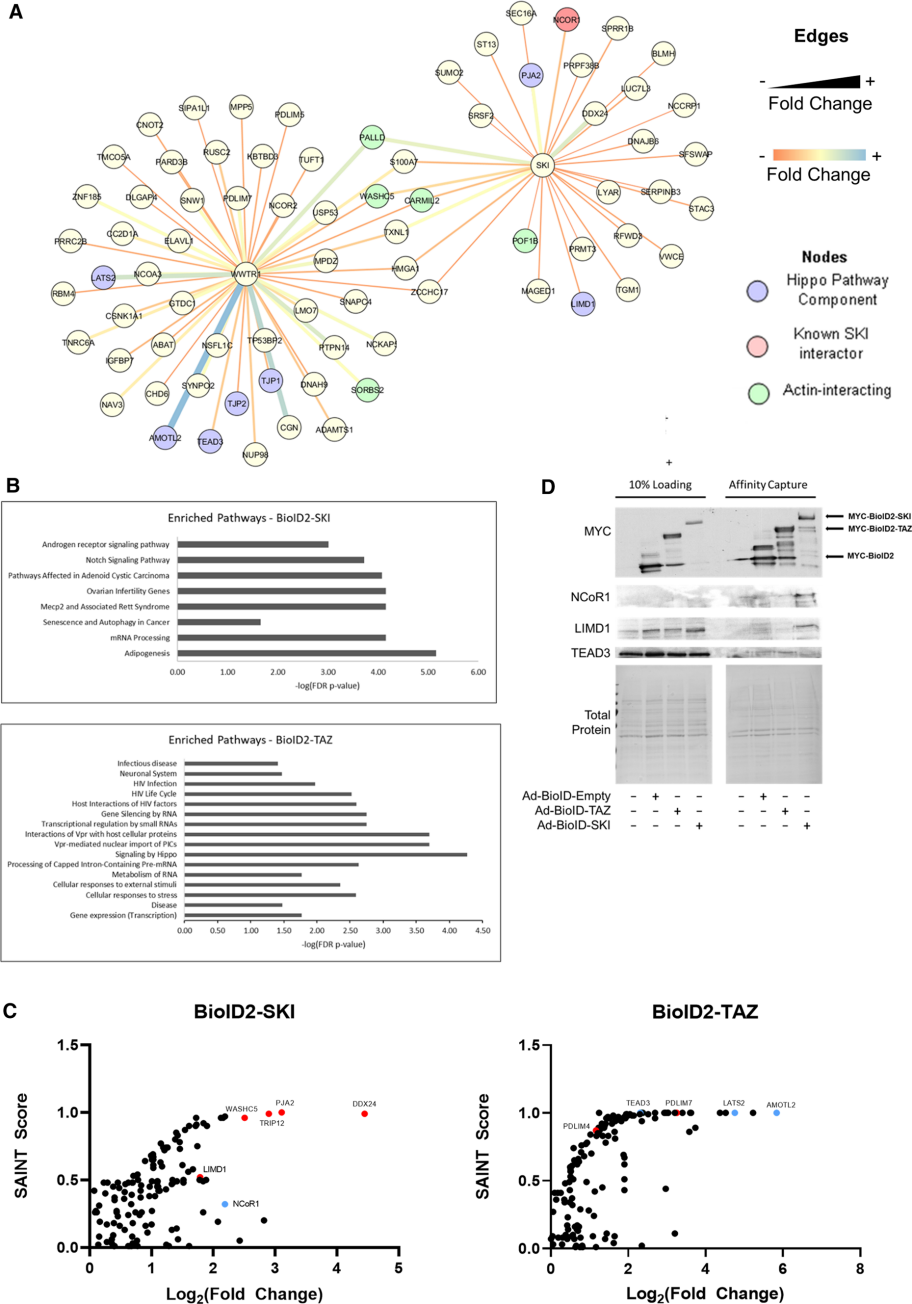
SKI and TAZ interactomes overlap in primary human cardiac fibroblasts. Primary human cardiac fibroblasts were infected with adenovirus constructs overexpressing MYC-BioID2 fusion proteins (TAZ or SKI) or empty MYC-BioID2 for 24 h. Cell cultures were then supplemented with 20 µM biotin, and incubated for another 24 h prior to harvesting. Untreated controls were also included to exclude endogenously biotinylated proteins. **a** Graphical representation of the TAZ (WWTR1) and SKI interactomes in human cardiac fibroblasts. Edge thickness and color is representative of the fold-change enrichment of the prey obtained by affinity capture. Hippo pathway components are highlighted in violet, while known SKI interactors are highlighted in red. **b** Pathway enrichment analysis for both SKI and TAZ interactomes. **c** Plotting of SAINT scores versus log2 fold-change enrichment of potential interactors. A SAINT score closer to 1 indicates greater likelihood of interaction. Select known interactors are indicated in blue, while novel interactors are indicated in red. **d** Immunoblotting was used to confirm novel interaction between SKI and LIMD1. Data shown are representative of *n* = 4 biological replicates

The SKI interactome consisted of 32 potential interactors, while the TAZ interactome contained 53 (Fig. 7a). Previously published interactors for both SKI (e.g., NCoR1) and TAZ (e.g., TEAD3) were used to confirm the validity of experiments, and verified by Western blot (Fig. 7b, d). We interrogated both interactomes against each other and found several interactors in common, 3 of which are associated with actin cytoskeleton dynamics: Palladin (PALLD), WASH Complex Subunit 5 (WASHC5, formerly KIAA0196), and Capping Protein Regulator and Myosin 1 Linker 2 (CARMIL2, formerly RLTPR). Pathway analysis of the SKI interactome indicated modest pathway enrichment for Notch signaling, adipogenesis, and senescence and autophagy (Fig. 7c). The TAZ interactome was most enriched for gene transcription, Hippo signaling, and cellular responses to stress and external stimuli. Within the SKI interactome, two candidate interactors became of interest, because they are suspected to regulate the Hippo pathway: PJA2 ubiquitin ligase and LIM Domain-containing protein 1 (LIMD1). LIMD1 was chosen for further investigation, because there is dearth of information regarding PJA2 and TAZ. Moreover, LIM proteins have been established as regulators of LATS1/2, and LIMD1 is specifically required to inhibit and sequester LATS1/2 at focal adhesions or tight junctions in other cell types [21, 23].

### LIMD1 mediates SKI’s effects on TAZ

**Fig. 8:**
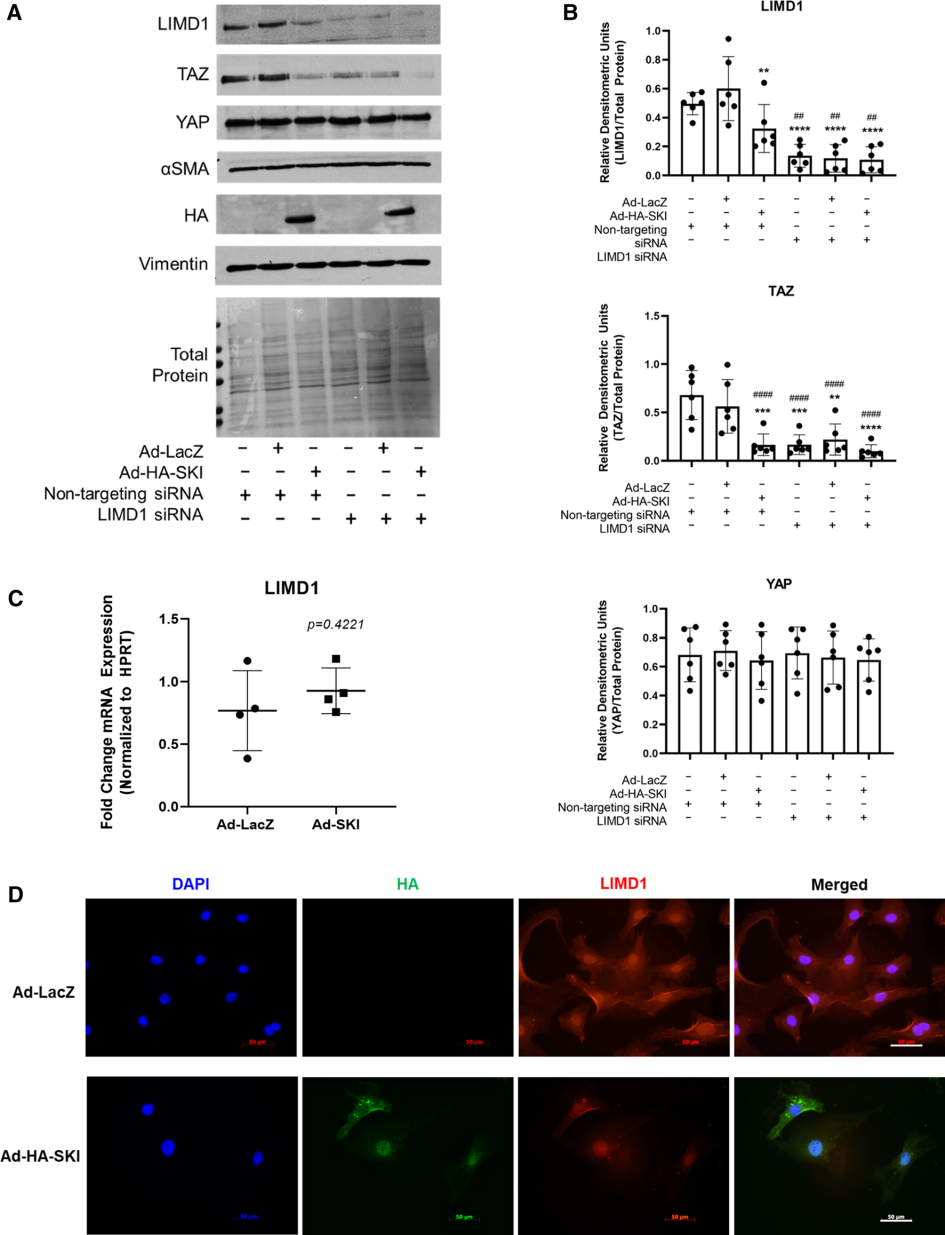
TAZ expression is regulated by LIMD1 in cardiac myofibroblasts. **a** Immunoblotting of whole cell lysates from activated (P1) primary rat cardiac myofibroblasts transfected with siRNA targeting *Limd1* 24 h prior to infection with Ad-HA-SKI or Ad-LacZ control. A non-targeting siRNA pool functioned as a control. **b** Quantification of data shown in A, with *n* = 6 biological replicates. **P < 0.01, ****P < 0.001 when compared to Ad-LacZ infected controls; ^##^*P* < 0.01, ###*P* < 0.001, ####*P* < 0.0001 when compared to cells only treated with non-targeting siRNA pool. **c**, **d** P1 primary rat cardiac myofibroblasts were cultured on stiff plastic (C) or glass (D) surfaces and infected with SKI-expressing adenovirus (Ad-HA-SKI) for 36 h. mRNA was isolated and (C) qRT-PCR of *Limd1* was performed on *n* = 4 biological replicates. Fixed cells (D) were probed for LIMD1 (red) and HA-SKI (green) by indirect immunofluorescence, with nuclei counterstained with DAPI (blue). Scale bar = 50 µm. Images are representative of *n* = 3 biological replicates, with 2 technical replicates each. Data shown in B and C are reported as the mean ± SD

LIMD1 has not been previously examined in the context of regulation of cardiac fibroblast activation. Therefore, we queried LIMD1 in primary rat cardiac myofibroblasts in vitro using both SKI overexpression and RNAi-mediated *Limd1* knockdown. The expression of LIMD1 protein in cardiac myofibroblasts was unaffected by neither Ad-LacZ, nor non-targeting siRNA pools (Fig. 8a, b). However, there was a notable decrease in endogenous LIMD1 expression with SKI overexpression, suggesting that whatever interaction exists between the two, it promotes the downregulation of LIMD1 protein expression. *Limd1* knockdown mimicked SKI overexpression in that only TAZ was apparently affected by decreased LIMD1 expression, whereas YAP expression remained entirely unaltered. When probing for LIMD1 by immunofluorescence, there was a decrease of its the staining between SKI-overexpressing and Ad-LacZ infected controls (Fig. 8d), with a notable shift from cytosolic to nuclear expression. There was no overt co-staining of SKI and LIMD1 in the cytosol; however, the nucleus was positively stained for both proteins. Finally, when looking at mRNA transcription with SKI overexpression, there was no significant change in *Limd1* transcription (Fig. 8c), suggesting that SKI may indeed interact with LIMD1 protein to downregulate its function or activity in cardiac myofibroblasts.

## Discussion

**Fig. 9:**
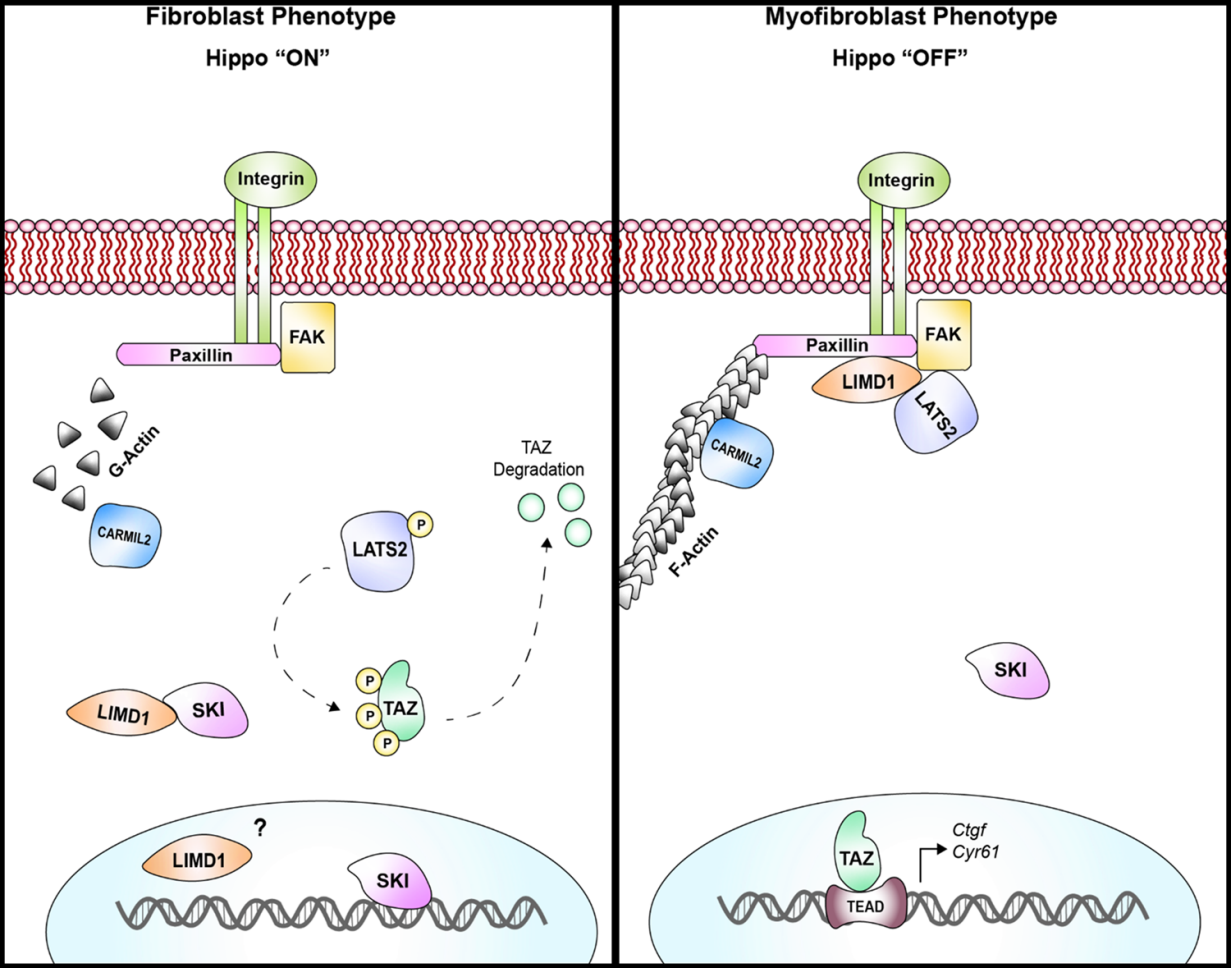
Model of SKI-mediated regulation of Hippo signaling and cardiac fibroblast activation. When SKI is localized in the cytoplasm, LIMD1 can freely associate and inhibit the function of LATS2 kinase, thus allowing TAZ-dependent, pro-fibrotic signaling to occur. Conversely, when SKI is functioning in the nucleus, it inhibits LIMD1 which, in turn, de-represses LATS2 kinase. The result is the phosphorylation and proteasomal degradation of TAZ, and the inhibition of the activated myofibroblast phenotype

The activation of cardiac fibroblasts to hyper-secretory, hyper-proliferative myofibroblasts is a phenomenon wherein the subcellular mechanisms are still poorly understood. Initially triggered by the release of cytokines after myocardial injury (i.e., MI, hypertension, diabetes), cardiac fibroblast activation is the primary event necessary for physiological wound healing; however, its chronic stimulation promotes cardiac fibrosis and ultimately leads to heart failure. Here, we demonstrate that Hippo signaling is a modulator of cardiac fibroblast physiology and that it is positively regulated by SKI. SKI is a protein that is typically regarded solely as an inhibitor of SMAD-dependent TGF-β signaling. Our findings suggest that SKI interacts with—and likely inhibits—LIMD1, which then de-represses the LATS2 kinase; this, in turn, enables LATS2 to specifically phosphorylate and cause the proteasomal degradation of TAZ (Fig. 9). Furthermore, we found that LATS2 interacts with TAZ whereas LATS1 does not (Fig. 7), and that LATS2 knockdown prevented TAZ downregulation with SKI overexpression (Fig. 5e). The signaling cascade results in a decrease in myofibroblast markers, and promotion of the quiescent fibroblast phenotype.

Our prior studies demonstrate that SKI is dysregulated (i.e., sequestered to the cytosol), during post-MI cardiac remodeling and fibrosis and functions to downregulate the expression of αSMA and ED-An FN [11, 12] in primary cardiac myofibroblasts. It was also established that SKI overexpression in primary cardiac myofibroblasts upregulates MMP-9 transcription and secretion [31], and its chronic expression represses autophagy-mediated survival responses which normally enable the pro-fibrotic phenotype to persist [60]. Recently it was found that SKI functions to inhibit pro-fibrotic signaling in cardiac myofibroblasts via SMAD-independent MAPK signaling [61], suggesting that its functions are far more pleiotropic than initially described. We demonstrate for the first time that SKI activates Hippo signaling in cardiac myofibroblasts and does so in a manner than only targets TAZ, and not YAP. We have also observed that TAZ expression is significantly upregulated in the expanding infarct scar during chronic post-MI remodeling, while increased YAP expression is distinctly observable in the vasculature; this is a compelling observation as YAP and TAZ are often described as having the same or redundant functions in a given pathology. We suggest that while YAP does possess some pro-fibrotic properties and is the more routinely examined effector of the Hippo pathway, TAZ is possibly more closely implicated in cardiac fibroblast activation and post-MI fibrosis. Our Western data indicate coincident expression of TAZ and POSTN in both viable (surviving) left ventricular myocardium and in the infarct scar of post-MI rat hearts, and accompanying immunofluorescence studies shows some scattered TAZ expression in post-MI hearts is localized to the infarct scar, with relatively intense staining in medial layer of the vasculature in the myocardium adjacent to the infarct scar and in the cardiomyocytes of the border zone surviving myocardium (Fig. 4b, and Supplemental Fig. 5). Thus the increase in TAZ observed in these tissues may be a combination of myofibroblast expression, activated vascular smooth muscle and surviving cardiomyocytes in the myocardium bordering the infarct scar.

Recent investigations have addressed the role of the Hippo pathway in the context of post-MI cardiac fibrosis. Using *Tcf21*^MerCreMer^*-Lats1/2*^ fl/fl^ model, Xiao et al. demonstrated that not only did fibroblast-specific deletion of both the LATS kinases yielded spontaneous myocardial fibrosis, but also exacerbated the severity of post-MI scar formation [56]. Although the study also included an examination of the post-MI response in *Tcf21*^MerCreMer^*-Lats1/2*^ fl/fl^*-Yap*^fl/fl^ mice as well, it is unclear whether the combined deletion of *Lats1*, *Lats2*, and *Yap* in cardiac fibroblasts markedly reduced the degree to which replacement fibrosis expanded compared to controls. In addition, RNAseq and chromatin occupancy studies indicated that in the absence of LATS1/2, YAP activation promotes a shift in ECM-related gene expression, and upregulates the proto-oncogene c-MYC. While these findings are valuable to understanding YAP activation, the data were acquired in immortalized NIH-3T3 fibroblasts, rather than in primary cells [56]. Despite these findings, there are no data in the literature regarding the expression of TAZ in concert with *Lats1/2* deletion, an experiment which would once again clarify whether YAP and TAZ are indeed redundant or independent in the context of cardiac fibrosis. It would be of interest to determine whether the observed paradigm also exists within activated myofibroblasts, as the model employed the *Tcf21* promoter, known only to be active in the quiescent fibroblast phenotype [26, 51].

While it has been argued that TAZ plays a redundant role in cardiomyocyte survival and proliferation with respect to YAP [55], we postulate that TAZ may target and upregulate a unique pro-fibrotic gene program during post-MI wound healing. Studies in other fibrotic pathologies have previously reported disparate expression patterns between YAP and TAZ, suggesting that these paralogs should not always be considered functionally linked. For example, work by Liu et al. to investigate idiopathic pulmonary fibrosis (IPF) indicate that TAZ upregulation exceeds that of YAP in IPF tissue, as its expression is predominantly nuclear in activated fibroblasts within diseased samples [35]. Similar results have been confirmed by other groups [39], and are echoed in studies of chronic kidney disease and renal fibrosis [1, 38]. Our data are in agreement with these findings, but also implicate SKI in the specific regulation of TAZ in the context of tissue fibrosis. As the current literature is primarily focused on YAP as the main nuclear Hippo pathway effector, and tendency to refer to YAP and TAZ interchangeably, it will be of benefit for future investigators to use a design which includes independent examination of these paralogs as separate entities with unique, and likely cell-specific functions. The evidence presented in the current study was obtained using constitutively active variants of YAP and TAZ, which yielded similar results for both paralogs in overexpression studies and thus the likelihood for the existence of further intracellular, extracellular, and matrix-mediated signaling to promote or deter YAP/TAZ activation in cardiac fibrosis is high.

We carried out BioID2 analyses to address novel mechanisms by which SKI interacts, and potentially regulates, Hippo signaling in primary cardiac fibroblasts. Previous oncological studies suggest that SKI interacts with several core Hippo pathway components, including LATS2; however, they utilized cancer cell lines (i.e., breast, kidney, and lung) [40, 58]. They revealed a decrease in TAZ protein expression with ectopic SKI expression, as well as a marked reduction in cell proliferation, migration, and tumorigenicity. The work done by Rashidian et al*.* suggests that SKI directly interacts with several components of the Hippo pathway (e.g., LATS2, MOB1, MOB2) [40]. We point out that these results were obtained outside the context of genuine pathology, as they were conducted in HEK 293 T cells overexpressing both the bait and prey proteins. Several other groups have remarked that physiological SKI functionality is not observable in most cell lines, and therefore, those studies should be cautiously interpreted [5, 6, 48, 52]. Several reports indicate that SKI’s functions are often both cell- and pathology-specific (as reviewed by Tecalco-Cruz and colleagues [52]), and this is especially evident when examining results from immortalized cells. To first demonstrate the efficacy of the BioID2 system, we captured the interactome of both SKI in HEK 293A cells, and found that the hits acquired from the test largely corroborated the literature (Supplemental Fig. 2a). However, when comparing this SKI interactome to that captured from primary human cardiac fibroblasts, only three interactors overlap among them (Supplemental Fig. 2b). With respect to the cardiac fibroblast SKI interactome, we specifically focused on LIMD1 as a point of interaction between SKI and Hippo signaling as it had already been implicated in the modulation of LATS-dependent Hippo inhibition. Additionally, when considering the TAZ cardiac fibroblast interactome, several LIM domain proteins were identified as potential points of interaction (Fig. 6A), including PDLIM5, suggesting that there may be another measure of TAZ regulation that has yet to be described.

While several LIM domain protein family members have been identified, their role in cell physiology and pathophysiology remains largely unknown. There is some evidence that LIMD1 functions as a scaffold for protein–protein interactions [16], and may play a role in cytoskeletal organization [2]. LIMD1 has been reported to inhibit the activity of LATS kinases by sequestering them to the cytosol and at focal adhesions and junctional complexes [21, 23]. Work by Jagannathan et al*.* also demonstrated that LIMD1 modulated YAP activity in proliferating epithelial cells, and that this regulatory process functions independently of substrate or ECM stiffness [23]. This notwithstanding, the majority of these studies were conducted in cell lines, and may not accurately reflect physiologic LIMD1 regulation. We observed a decrease in LIMD1 expression upon induction of SKI in vitro (Fig. 8B), which coincides with a decrease in actin polymerization and stress fiber formation. Previous studies in other mesenchymal cells have shown that LIMD1 expression is reduced by treatment with the myosin-II inhibitor, blebbistatin [20, 43]. It is possible that SKI’s effects on the actin cytoskeleton may be more direct in generating the downregulation of LIMD1. Other studies in PDZ-LIM proteins have demonstrated that their association with the CARMIL2 (formerly Arp2/3) actin-nucleating protein dictates stress fiber cross-linking [36]. This is of note as we isolated CARMIL2 as a potential SKI interactor in the BioID2 assay. SKI’s interaction or inhibition of LIMD1 may be in a complex with CARMIL2, although this has yet to be confirmed. Despite the current lack of evidence directly linking TAZ and LIMD1, our findings indicate that LIMD1 is a key inhibitor of the TAZ-Hippo pathway in cardiac fibroblasts, and that its absence yields a similar cellular response as does SKI overexpression.

## Conclusions

SKI is an anti-fibrotic protein whose function(s) may extend beyond canonical TGF-β1 signaling. We elucidate novel molecular mechanisms governing cardiac fibroblast phenotype modulation, and these findings warrant further investigation of Hippo signaling and its therapeutic targeting within the context of cardiac fibrosis. Our current results improve our understanding of the role of TAZ and YAP as stand-alone, distinct factors in fibrogenesis. The data also present a novel mechanism of action for SKI, which is intimately linked to the actin cytoskeleton. Future studies to examine LIMD1 and its function in cardiac fibroblasts in combination with SKI’s role in fibroblast physiology may lead to the generation of data that could be exploited for selective therapeutic targeting in cardiac fibrosis and heart failure.

## Supplementary Information

Below is the link to the electronic supplementary material.

Electronic supplementary material 1 (XLS 162 kb)

Electronic supplementary material 2 (XLS 125 kb)

Electronic supplementary material 3 (XLS 149 kb)

Electronic supplementary material 4 (XLS 144 kb)

Electronic supplementary material 5 (XLS 78 kb)

Electronic supplementary material 6 (XLS 78 kb)

Electronic supplementary material 7 (XLS 94 kb)

Electronic supplementary material 8 (XLS 81 kb)

Electronic supplementary material 9 (TIF 1120 kb)

Electronic supplementary material 10 (TIF 2073 kb)

Electronic supplementary material 11 (TIF 1420 kb)

Electronic supplementary material 12 (TIF 3417 kb)

Electronic supplementary material 13 (TIF 1497 kb)

## Data Availability

The data sets used and/or analyzed during the current study are available from the corresponding author on reasonable request. The mass spectrometry data are available as part of the online data supplement, as well as on the ProteomeXchange PRIDE partner repository, as described in the methods section.

## Abbreviations

αSMA: Alpha-Smooth Muscle Actin
AP-1: Activator Protein-1
β-TrCP1: Beta-Transducin repeat Containing Protein 1
CARMIL2: Capping Protein Regulator and Myosin 1 Linker 2
CK1: Casein Kinase 1
CTGF: Connective Tissue Growth Factor
CTHRC1: Collagen Triple Helix Repeat Containing 1
CYR61: Cysteine-Rich angiogenic inducer 61 (also called CCN1)
ECM: Extracellular Matrix
ED-A: Extracellular Domain-A
LATS1/2: Large Tumor Suppressor 1 or 2
LIMD1: LIM Domain-containing protein 1
MST1/2: Macrophage-Stimulating 1 or 2
NCoR1: Nuclear Co-Repressor 1
POSTN: Periostin
RLTPR: RGD, Leucine-Rich Repeat, Tropomodulin And Proline-Rich-Containing Protein
SMAD: Mothers Against Decapentaplegic homolog
TAZ: Transcriptional co-Activator with PDZ-binding motif (also called WWTR1)
TEAD: TEA Domain family member
TEF: Transcriptional Enhancer Factor
WASHC5: WASH Complex Subunit 5
WWTR1: WW domain-containing Transcription Regulator protein 1 (also called TAZ)
YAP: Yes-Associated Protein
