# Supplementary figures and images for: SKI activates the Hippo pathway via LIMD1 to inhibit cardiac fibroblast activation
Source: Basic Res Cardiol. 2021 Apr 13;116(1):25. doi: 10.1007/s00395-021-00865-9 (PMC8043893; doi:10.1007/s00395-021-00865-9)

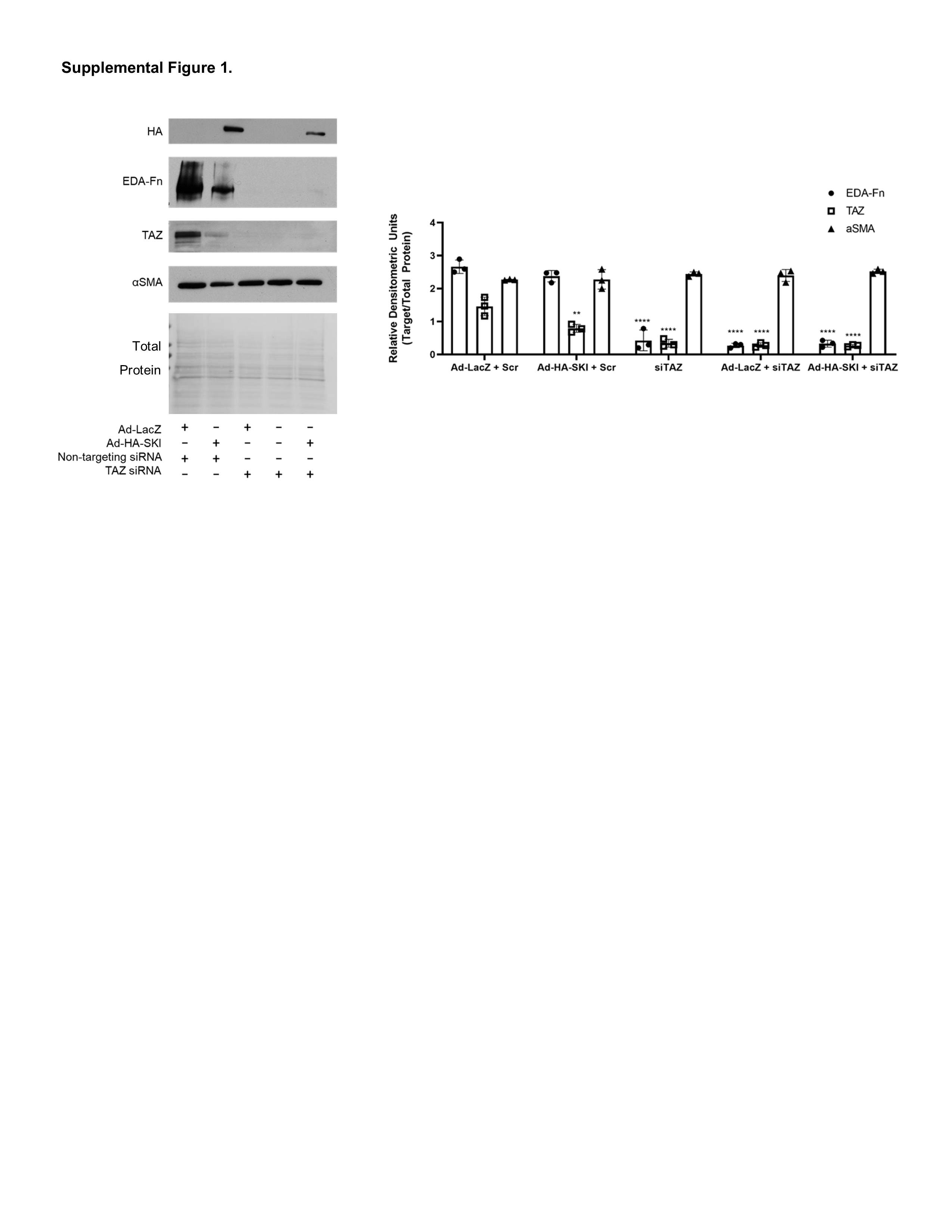

Supplement: Supplementary file 9 — Electronic supplementary material 9 (TIF 1120 kb) [file 395_2021_865_MOESM9_ESM.tif]

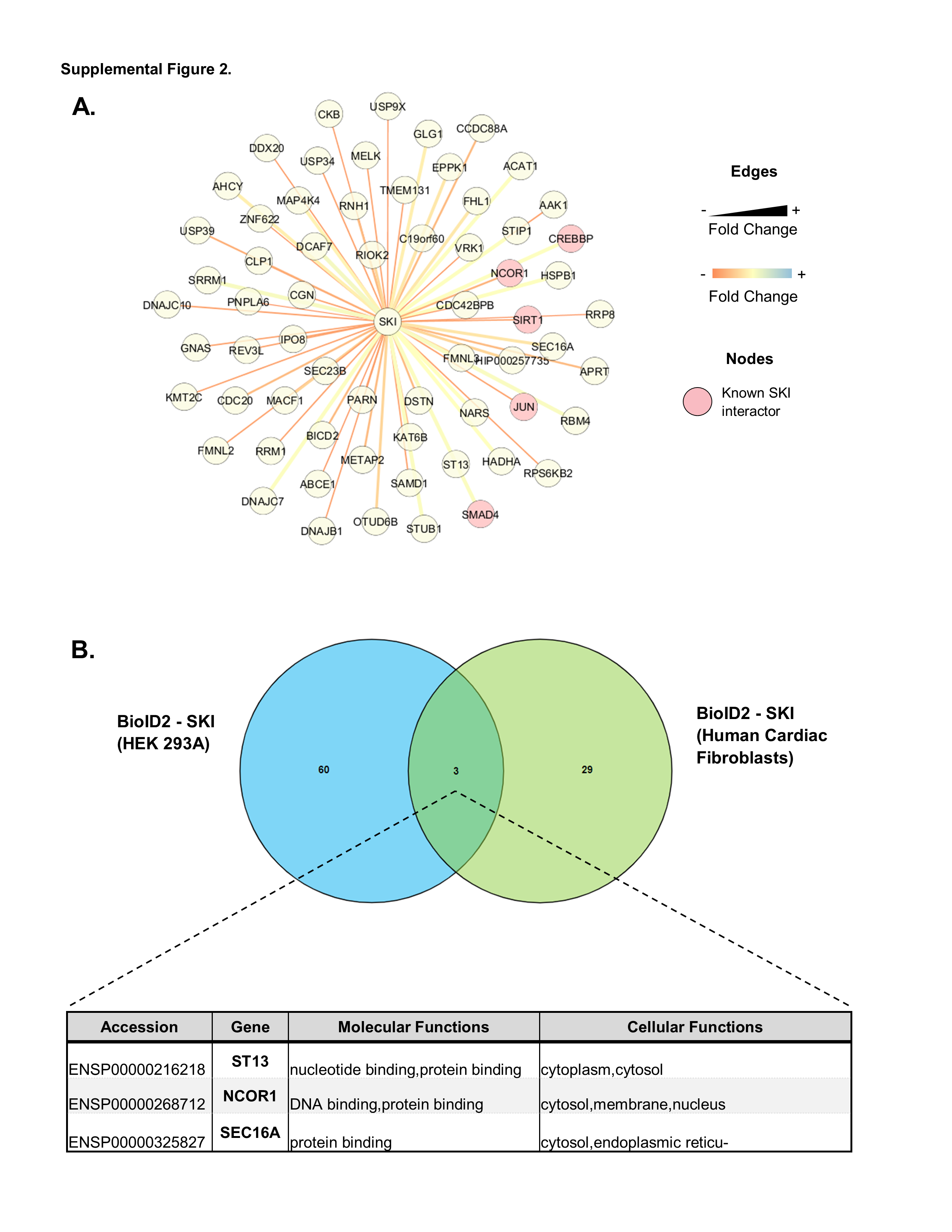

Supplement: Supplementary file 10 — Electronic supplementary material 10 (TIF 2073 kb) [file 395_2021_865_MOESM10_ESM.tif]

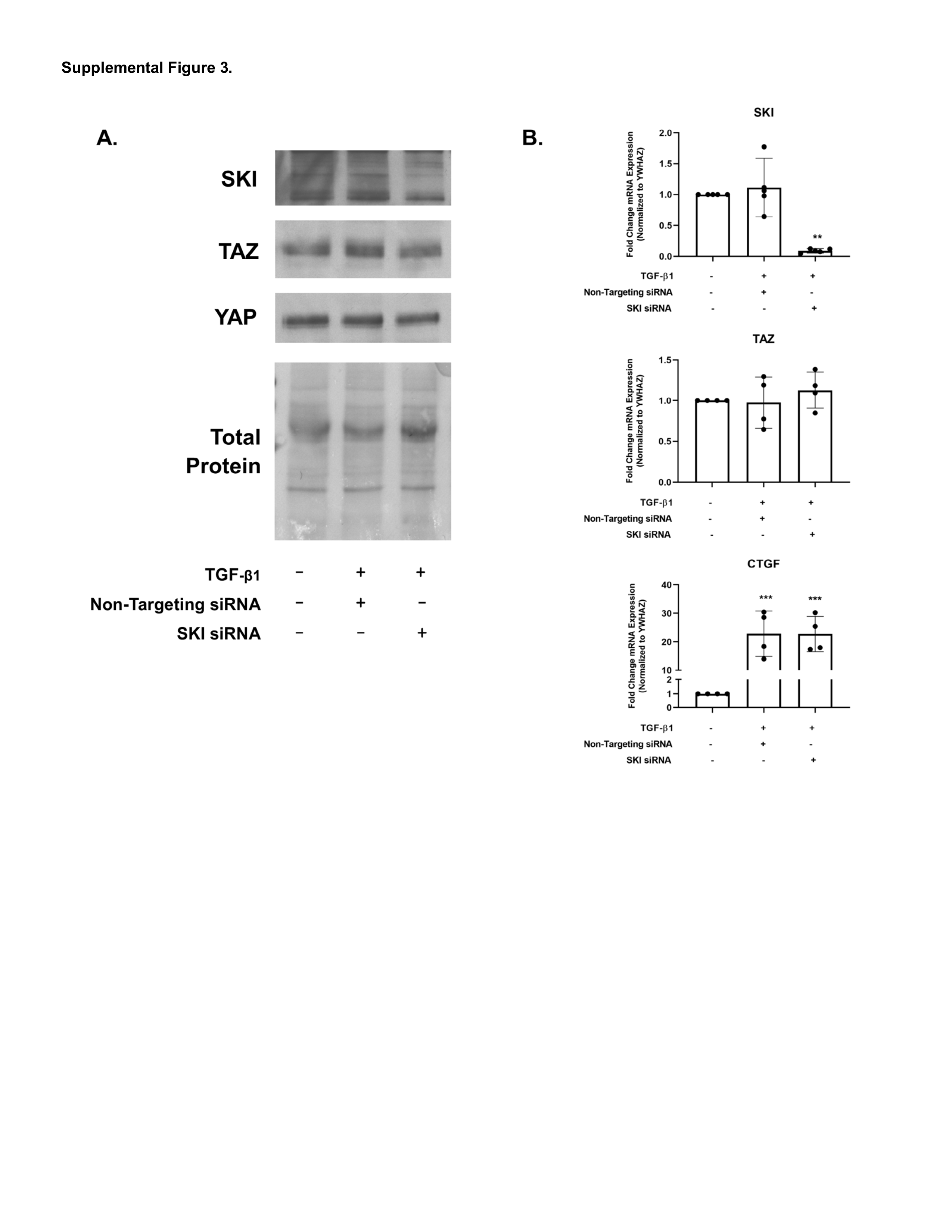

Supplement: Supplementary file 11 — Electronic supplementary material 11 (TIF 1420 kb) [file 395_2021_865_MOESM11_ESM.tif]

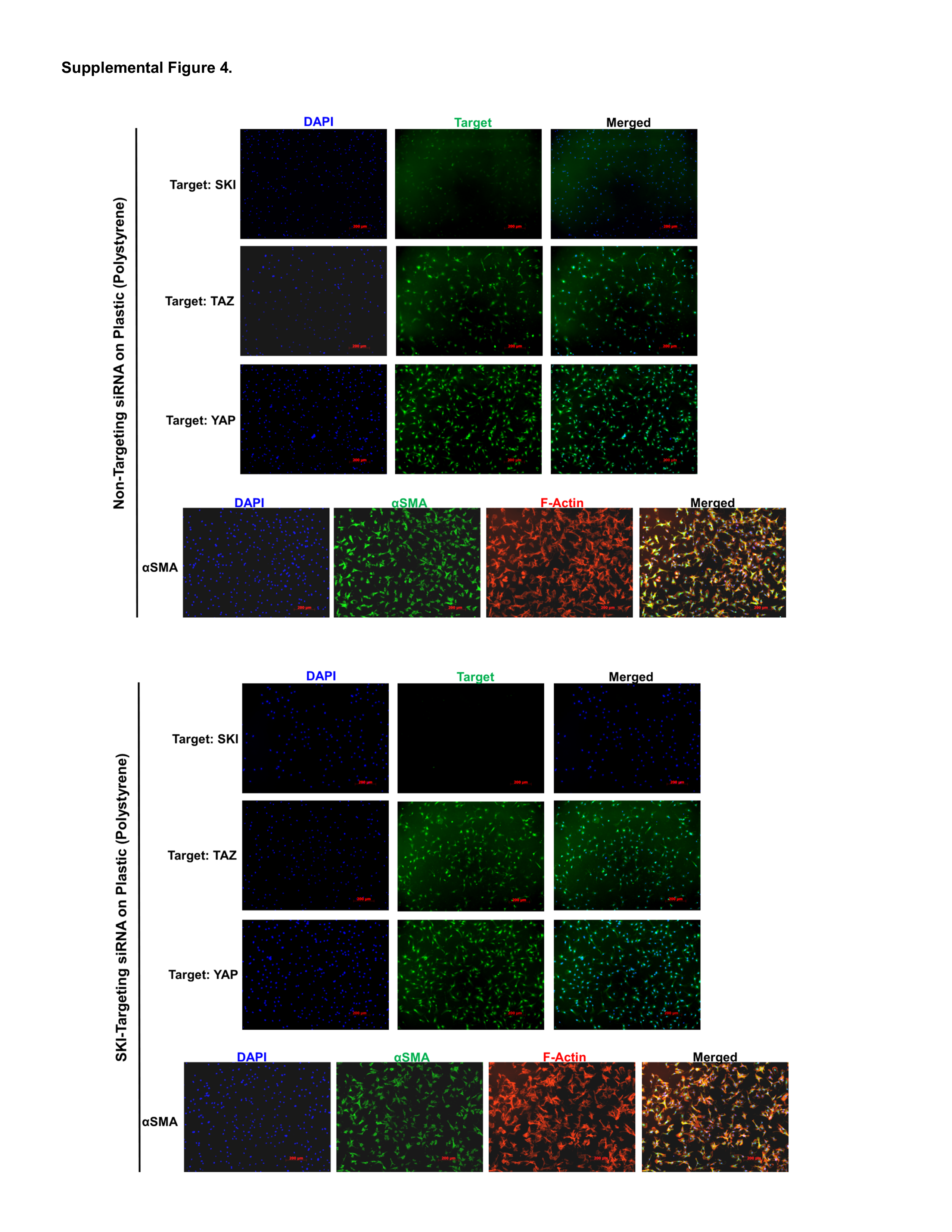

Supplement: Supplementary file 12 — Electronic supplementary material 12 (TIF 3417 kb) [file 395_2021_865_MOESM12_ESM.tif]

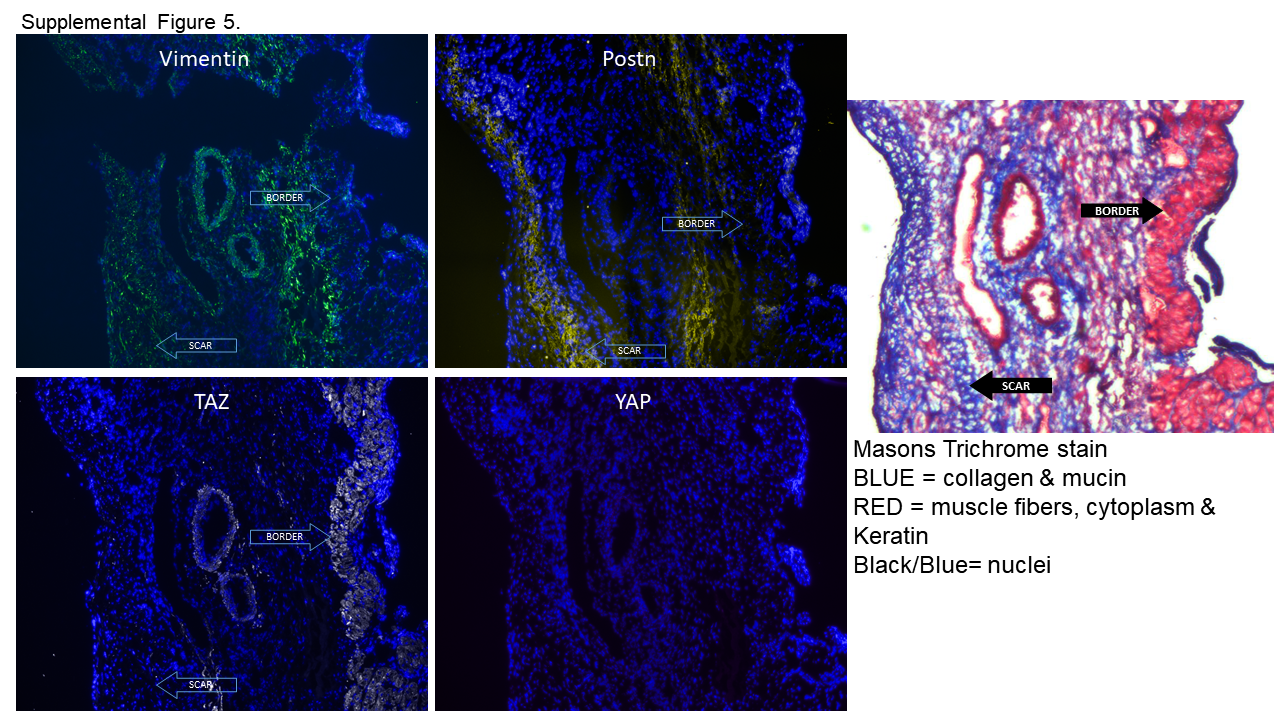

Supplement: Supplementary file 13 — Electronic supplementary material 13 (TIF 1497 kb) [file 395_2021_865_MOESM13_ESM.tif]
